# Co-optimization of therapeutic antibody affinity and specificity using machine learning models that generalize to novel mutational space

**DOI:** 10.1038/s41467-022-31457-3

**Published:** 2022-07-01

**Authors:** Emily K. Makowski, Patrick C. Kinnunen, Jie Huang, Lina Wu, Matthew D. Smith, Tiexin Wang, Alec A. Desai, Craig N. Streu, Yulei Zhang, Jennifer M. Zupancic, John S. Schardt, Jennifer J. Linderman, Peter M. Tessier

**Affiliations:** 1 Department of Pharmaceutical Sciences, University of Michigan, Ann Arbor, MI 48109 USA; 2 Biointerfaces Institute, University of Michigan, Ann Arbor, MI 48109 USA; 3 Department of Chemical Engineering, University of Michigan, Ann Arbor, MI 48109 USA; 4 Department of Chemistry, Albion College, Albion, MI 49224 USA; 5 Department of Biomedical Engineering, University of Michigan, Ann Arbor, MI 48109 USA

**Keywords:** Antibody therapy, Machine learning, Protein design

## Abstract

Therapeutic antibody development requires selection and engineering of molecules with high affinity and other drug-like biophysical properties. Co-optimization of multiple antibody properties remains a difficult and time-consuming process that impedes drug development. Here we evaluate the use of machine learning to simplify antibody co-optimization for a clinical-stage antibody (emibetuzumab) that displays high levels of both on-target (antigen) and off-target (non-specific) binding. We mutate sites in the antibody complementarity-determining regions, sort the antibody libraries for high and low levels of affinity and non-specific binding, and deep sequence the enriched libraries. Interestingly, machine learning models trained on datasets with binary labels enable predictions of continuous metrics that are strongly correlated with antibody affinity and non-specific binding. These models illustrate strong tradeoffs between these two properties, as increases in affinity along the co-optimal (Pareto) frontier require progressive reductions in specificity. Notably, models trained with deep learning features enable prediction of novel antibody mutations that co-optimize affinity and specificity beyond what is possible for the original antibody library. These findings demonstrate the power of machine learning models to greatly expand the exploration of novel antibody sequence space and accelerate the development of highly potent, drug-like antibodies.

## Abstract (web summary)

Optimising antibody properties such as affinity can be detrimental to other key properties. Here the authors use machine learning to simplify the identification of antibodies with co-optimal levels of affinity and specificity for a clinical-stage antibody that displays high levels of on- and off-target binding.

## Introduction

Antibody therapeutics are being used to treat human disorders ranging from cancer and autoimmune diseases to allergies and neurodegenerative diseases^1–3^. The success of antibody therapeutics is due in large part to their highly attractive molecular properties, including their high affinities, long half-lives, potent effector functions, and excellent biophysical properties (e.g., high stability and solubility)^4–6^. However, it is well known that antibody candidates selected from immunization or in vitro library sorting typically have a wide range of biophysical properties, such as diverse solubilities and viscosities in common formulation conditions^7–14^. In many cases, antibody candidates with the highest bioactivities exhibit one or more undesirable biophysical properties that impede production, formulation and/or delivery. This is often discovered late in the developmental process, after substantial investment of limited resources, and can compromise the therapeutic potential of otherwise promising candidates^9,12,15^. Therefore, there is a need for antibody engineering methods to improve their biophysical properties while maintaining high affinity and bioactivity, especially during early stages of development. Unfortunately, it is commonly observed that improving a given sub-optimal antibody property, such as specificity or solubility, results in deficits in other properties, such as affinity^16–22^. These strong tradeoffs are often due to the central role of the complementarity-determining regions (CDRs) of antibodies in strongly impacting both their affinities and biophysical properties^18,23–25^.

Therefore, there is significant need for simple and robust methods for predicting CDR mutations that co-optimize antibody affinity and various biophysical properties with minimal experimentation. Encouragingly, advances in high-throughput evaluation of antibody properties using a combination of display technologies (e.g., phage and yeast-surface display) and deep sequencing have enabled the generation of large datasets that link multisite antibody mutations, such as mutations in multiple CDRs, with different levels of a given property (e.g., high and low levels of affinity, stability or specificity)^26–32^. However, these datasets have been substantially underused due to shortcomings in extracting meaningful features from antibody primary sequences and incorporating them into models that accurately predict different levels of a given antibody property. Conventional methods of analyzing such deep sequencing datasets typically use frequencies or enrichment ratios to identify promising antibody mutants, but these approaches fail to make use of the large amount of available antibody sequence information and, in some cases, only result in modest ability to identify antibodies with large improvements in properties such as affinity^23,33^. These approaches are typically limited to the analysis of antibody variants observed in the sequenced libraries, which represent an extremely small fraction of maximal sequence space, even for antibody mutations only in the CDRs. An exception is a reported method that predicts enrichment ratios^31^.

One logical and simple approach for using the vast amount of sequence information obtained from antibody library deep sequencing is to encode the amino acid sequences as sequence-identity vectors (*e.g*., one-hot encoded antibody sequences) to build predictive models. Indeed, models developed using this approach are often highly accurate for predicting the classification of antibody properties, such as low or high affinity^34–36^. However, classification imposes binary labels on continuous properties, and intra-class variability such as *high* versus *very high* affinity is vital to antibody optimization. Therefore, it would be significant if methods could be developed for predicting continuous metrics that are strongly correlated with antibody properties based only on binary measurements of an experimentally tractable number of antibody sequences. Moreover, it would also be significant if such methods could generalize to novel mutational space, including at novel CDR sites not mutated in the original libraries, to greatly expand the fraction of the maximal sequence space explored during antibody optimization.

**Fig. 1:**
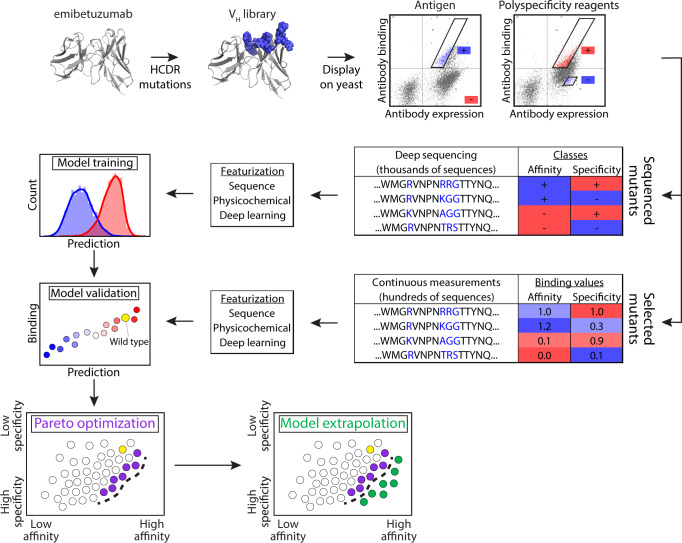
Overview of antibody library sorting, deep sequencing, and machine learning methods used to co-optimize the affinity and specificity of a therapeutic antibody. A clinical-stage antibody (emibetuzumab) was mutated at eight positions in three heavy chain CDRs, and the antibody libraries were sorted using yeast surface display and magnetic- and fluorescence-activated cell sorting for high affinity and high and low levels of non-specific binding. The sorted libraries were deep sequenced, and the resulting antibody sequences were used to train models for predicting metrics correlated with antibody affinity and specificity (non-specific binding) using different types of molecular features. These features included antibody sequences encoded as binary vectors, physicochemical features, and deep learning features. The resulting models were used not only to predict the classification of antibody affinity and specificity (e.g., high or low affinity), but also continuous metrics correlated with each property to predict intraclass variability (e.g., high vs. very high affinity). The model predictions were also used to identify antibody mutants in the library at the Pareto frontier that maximize antibody affinity to different extents while minimizing tradeoffs due to reduced specificity (i.e., increased non-specific binding). Some of the models, which generalized to novel mutational space, were used to identify antibodies with even greater improvements in affinity and specificity than was possible in the experimentally sorted libraries.

In this work, we have sought to address both outstanding challenges in order to co-optimize the affinity and specificity (non-specific binding) of a clinical-stage antibody (emibetuzumab^37^). This antibody is specific for c-Met [also known as tyrosine-protein kinase Met or hepatocyte growth factor receptor (HGFR)] and reached phase II clinical trials for the treatment of non-small cell lung cancer. Previous work has revealed that the high levels of non-specific binding for emibetuzumab are primarily due to the heavy chain CDRs^38^. Given that heavy chain CDRs also play a central role in mediating antibody affinity, we reasoned that CDR mutations in the heavy chain of emibetuzumab would exhibit strong tradeoffs between emibetuzumab on-target (affinity) and off-target (non-specific) binding. Therefore, we expected that co-optimized antibody variants with both high affinity and low non-specific binding would be extremely rare and require advanced methods to identify them. Moreover, we expected that it would be important to develop predictive models that can learn from large but sparsely sampled antibody CDR libraries and predict antibody properties for novel CDR mutants not sampled in the original libraries to identify rare co-optimized variants. Herein, we report an integrated experimental and computational approach that combines deep sequencing, machine learning, and high-throughput experimental methods to identify co-optimized therapeutic antibody variants, including variants with superior combinations of affinity and non-specific binding relative to a parental, clinical-stage antibody (Fig. 1).

## Results

### Conventional analysis of deep sequencing data is poorly predictive of co-optimal emibetuzumab variants

Toward our goal of identifying co-optimized emibetuzumab variants with high affinity and specificity (low non-specific binding), we designed a large antibody sub-library (~10^7^ variants) by mutating sites in the heavy chain CDRs that were predicted previously to mediate non-specific binding (Supplementary Fig. 1)^38^. We sampled the wild-type residue and five mutations at eight sites distributed across heavy chain CDRs 1 (one site), 2 (four sites) and 3 (three sites) that are predicted to reduce non-specific binding. Next, we displayed the library on the surface of yeast as single-chain Fab fragments and sorted the library via magnetic-activated cell sorting (MACS, rounds 1–2) against the antigen (HGFR) to remove fragmented or non-displaying antibodies. The MACS-sorted libraries were then sorted by fluorescence-activated cell sorting (FACS, round 3) for high levels of antigen binding and high and low levels of non-specific binding to two polyspecificity reagents (ovalbumin^39^ and soluble membrane proteins isolated from CHO cells^15,40,41^; Supplementary Fig. 2). Finally, we deep sequenced the input and FACS-sorted libraries and selected 4000 of the most frequently observed antibody mutants that were observed in both the affinity and specificity selections for further analysis (see Methods for more details).

To evaluate our ability to predict antibody mutants with high affinity and low levels of non-specific binding, we next Sanger sequenced 125 mutants from the FACS-sorted libraries and evaluated their relative levels of antigen and non-specific binding on the surface of yeast. Although it is logical to assume that antibody mutants which are most enriched (relative to the input) or most frequently observed will display superior properties, we found that this was generally not the case, in line with previous work^29,42,43^, although some exceptions have been observed^31,44^. We observed a lack of statistically significant positive correlations between antigen binding and frequency (Spearman’s ρ of 0.23 and *p* value of 0.08) or enrichment ratio (ρ of −0.01 and *p* value of 0.96) for positive antigen-binding selections. Moreover, we also observed a lack of statistically significant negative correlations between non-specific binding and frequency (ρ of −0.16 and *p* value of 0.14) for negative non-specific binding selections (Supplementary Fig. 3). While we did observe a significant negative correlation between non-specific binding and enrichment ratio (ρ of −0.47 and *p* value of 6 × 10^−6^), the lack of a corresponding significant correlation for affinity prevents the use of enrichment ratios for reliably identifying antibody variants that are co-optimal for both high affinity and specificity.

### Prediction of Pareto optimal antibody variants

**Fig. 2:**
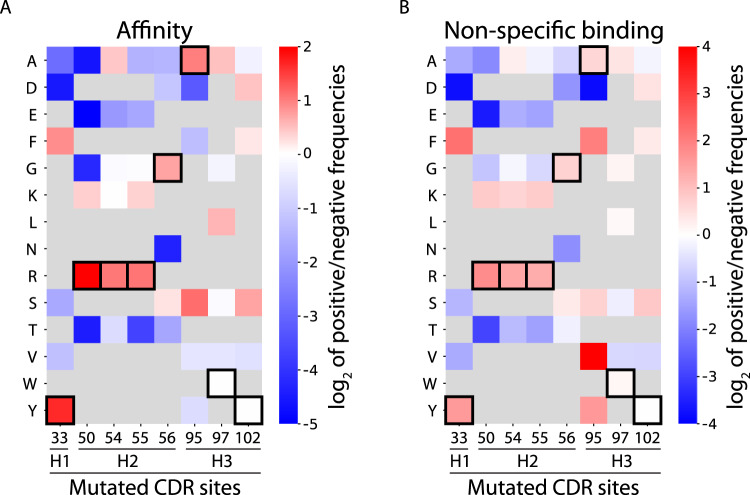
Levels of CDR residue enrichment in the sorted emibetuzumab libraries are similar for high affinity and high non-specific binding selections. Calculated ratios between the amino acid frequencies at each mutated site for library samples selected for (**A**) high affinity relative to low affinity and (**B**) high non-specific binding (ovalbumin and soluble membrane proteins) relative to low non-specific binding. Red mutations appeared more frequently in the high affinity and high non-specific binding library samples, while blue mutations appeared more frequently in the low affinity and low non-specific binding library samples. Wild-type residues are highlighted with black boxes, and residues that were not sampled in the libraries are indicated with gray shading.

We next evaluated the information contained in our selected dataset of 4000 sequences by analyzing the enrichment of library mutations in the positive sorts relative to the negative sorts (Fig. 2). We noticed strong enrichment of the wild-type residues for both the high affinity and high non-specific binding selections, particularly for Y33 in HCDR1 and R50 and R55 in HCDR2. Surprisingly, wild-type residues W97 and Y102 in HCDR3 were not enriched in the affinity selections, suggesting opportunities for further antibody affinity optimization.

To preserve this information for model development, we chose to encode the antibody V_H_ sequences as one-hot encoded binary vectors to capture the presence or absence of mutations at each site in our library. We hypothesized that classification algorithms with architectures that learn weights for these individual features would enable not only accurate predictions of property class (e.g., high vs. low affinity) but also accurate predictions of continuous property values (e.g., high vs. very high affinity).

**Fig. 3:**
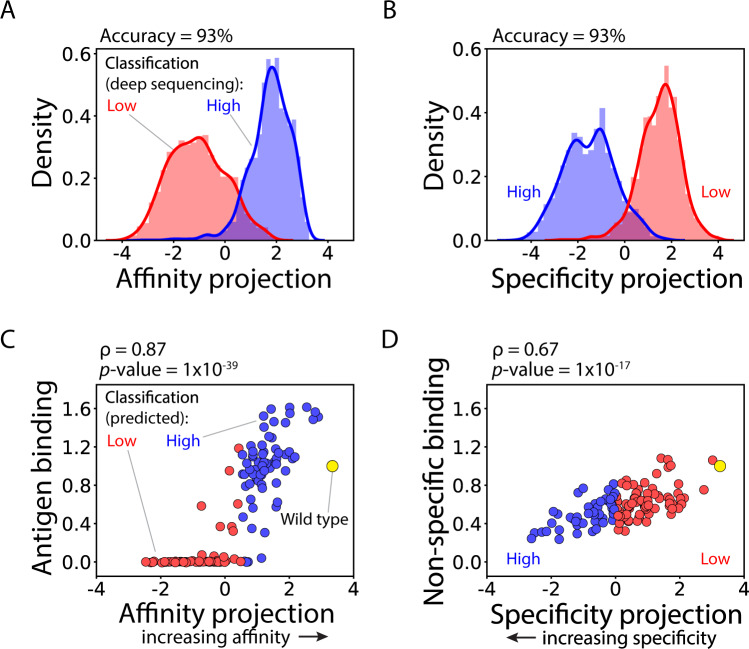
Models trained using supervised dimensionality reduction not only accurately classify antibody mutants with high and low levels of affinity and specificity but also accurately predict intraclass variability. **A**, **B** Linear discriminant analysis (LDA) models were trained using sequence-based features (one-hot encoded sequences as binary vectors) and displayed high accuracy for classifying antibody affinity and specificity for 4000 antibodies identified in the enriched libraries via deep sequencing. (C-D) The continuous predictions of the LDA models, which are referred to as projections, are strongly correlated with experimental measurements of the (**C**) relative affinity and (**D**) non-specific binding for 125 single-chain antibodies (Fabs) selected randomly from the sorted libraries. In (**C**), the antigen (HGFR) concentration was 1 nM, and the values are normalized between elotuzumab (value of zero) and wild-type emibetuzumab (value of one). In (**D**), the non-specific binding reagent was ovalbumin (0.1 mg/mL). In (**A**–**D**), the projection (x-axis) values that separated high and low classes (e.g., high and low affinity) were close to but not exactly zero. In (**C**) and (**D**), the experimental measurements are averages of two or three independent repeats. Independent two-sided *t*-tests were performed to determine significance.

To test this hypothesis, we evaluated the ability of relatively simple linear discriminant analysis (LDA) models to predict both antibody affinity and specificity (Fig. 3). LDA models predict binary classifications by first projecting high-dimensional data into a continuous one-dimensional space to optimize classification accuracy, and then finding the optimal classification cutoff within the one-dimensional space. We reasoned that the scalings learned by the models would reflect the different levels of mutational enrichment, and that the resulting one-dimensional feature-space predictions, referred to as projections, would correlate with the continuous values of antibody affinity and specificity that were measured experimentally.

Therefore, we trained LDA models to predict deep sequencing labels using the one-hot encoded features, herein referred to as OneHot models. The models were trained to predict each antibody property using five-fold cross-validation methods (80%:20% training:test data splits) to prevent overfitting. Encouragingly, the OneHot models strongly classified the affinity and specificity of the antibody mutants in the deep sequencing datasets, as both models resulted in 93% accuracy for classifying antibody affinity and specificity (Fig. 3A, B). These accuracies are similar to those obtained using a simpler classification algorithm (k-nearest neighbors) that makes predictions based on similarity to training set data alone (83% for affinity and 89% for specificity; Supplementary Fig. 4). This finding is consistent with our experience analyzing similar datasets with diverse types of models and demonstrates that classification of antibody properties such as affinity and specificity based on deep sequencing data is a relatively simple task and weakly dependent on the type of model used for the predictions.

However, predictions of property class (e.g., high vs. low affinity) are of limited use for identifying antibody mutants with the best combinations of properties, including affinity and specificity. Inspection of the LDA projections for each antibody property revealed both significant intraclass variability and that the values at either extreme predicted each property with the highest accuracies (Fig. 3A, B), suggesting that the LDA projections may be useful for predicting not only interclass differences (e.g., classification of low versus high affinity) but also intraclass differences (e.g., high versus very high affinity). Therefore, we also evaluated the ability of the model projections, which are continuous metrics and not actual property values, to describe the relative affinity and non-specific binding values for experimental measurements obtained using yeast surface display for the set of 125 antibody mutants that were isolated via Sanger sequencing after library sorting (Fig. 3C, D). None of these antibody mutants were present in the set of 4000 antibodies used for training and testing. Strikingly, we observed strong correlations between the model predictions and the experimental measurements, including Spearman correlation coefficients of 0.87 for affinity (*p* value of 10^−39^) and 0.67 for non-specific binding (*p* value of 10^−17^). These encouraging results indicate that continuous metrics correlated with each property can be predicted for the sequences in the library with relatively high accuracy, including for the 4000 sequences used in the training process.

The simplicity of the LDA models naturally raises the question of whether more complex machine learning models would lead to improved performance for predicting antibody affinity and specificity metrics. Therefore, we developed neural network models to predict affinity and specificity metrics. Each model included a dense layer with a single node to create final projections that could be used as metrics for correlation with each property and identification of optimal classification cutoffs (Supplementary Fig. 5). Notably, the neural network models performed similarly to the LDA models. The classification prediction accuracies were the same for both models for affinity and specificity, consistent with our general observation that the accuracy of classification of antibody properties based on deep sequencing data is weakly dependent on model complexity. For the prediction of continuous antibody properties, we observed the same performance for predicting antigen binding (Spearman's ρ of 0.87 for both models) and modestly improved performance for predicting non-specific binding (ρ of 0.70 compared to 0.67 for the LDA model).

**Fig. 4:**
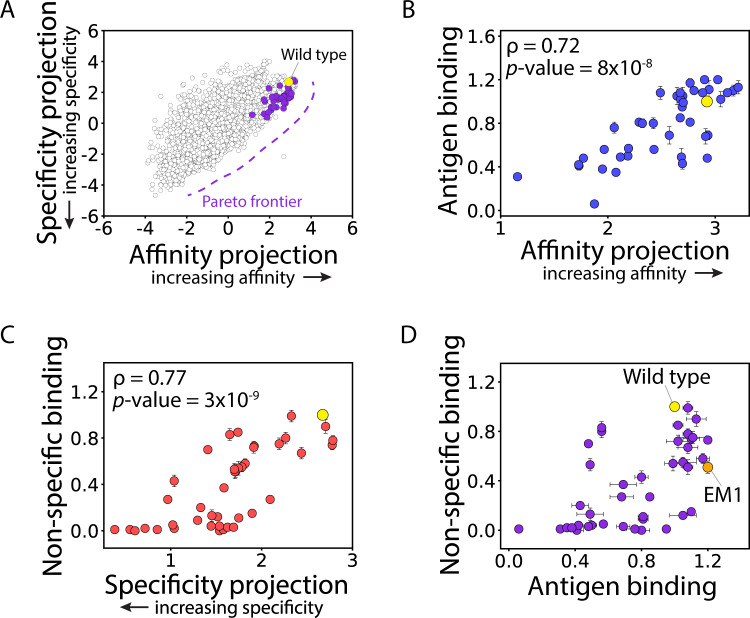
Model predictions and experimental evaluation of Pareto optimal affinities and specificities for emibetuzumab mutants in the sorted antibody libraries. **A** Visualization of the predicted Pareto frontier for the 4000 library variants using the LDA models reported in Fig. 3. Antibody mutants located along the Pareto frontier are predicted to display co-optimized affinity and specificity, as increases in either property requires decreases in the other one. **B**, **C** Antibody mutants (41 antibodies) at or near the predicted Pareto frontier were generated as soluble IgGs, and their (**A**) antigen binding (1 nM HGFR) and (**B**) non-specific binding (0.1 mg/mL ovalbumin) were evaluated and plotted relative to the continuous model predictions. **D** Comparison of the experimentally measured values of antigen and non-specific binding for the 41 IgGs reported in parts (**B**) and (**C**). In (**B**–**D**), the experimental measurements are averages of three independent repeats, and the error bars are standard deviations. Independent two-sided *t*-tests were performed to determine significance.

Given the strong performance of simple and easy-to-implement LDA models, we next plotted the LDA model projections of affinity and specificity for each of the 4,000 antibody sequences to directly visualize the tradeoffs between the two properties on a continuous scale (Fig. 4A). Notably, the emibetuzumab variants showed strong tradeoffs between both properties, as increases in affinity typically required reductions in specificity and vice versa. This analysis also revealed the Pareto frontier for the antibody library, which corresponds to the set of co-optimal antibody variants with the maximum affinities at each level of specificity. Notably, the wild-type antibody is not at the Pareto frontier and, therefore, it may be possible to increase both affinity and specificity at the same time.

To evaluate the predictions of Pareto optimal antibody variants, we next identified and produced 41 antibody mutants (as soluble IgGs) that were predicted to be at or near the Pareto frontier (Fig. 4A) and experimentally evaluated their levels of antigen (Fig. 4B) and non-specific (Fig. 4C) binding. Encouragingly, we find that the model predictions are strongly predictive of affinity (Spearman’s ρ of 0.72 and *p* value of 8 × 10^−8^; Fig. 4B) and specificity (ρ of 0.77 and *p* value of 3 × 10^−9^; Fig. 4C), which is not observed for conventional analysis using enrichment ratios and frequencies of the antibody mutants (Supplementary Fig. 6). Moreover, we find that simultaneous improvement of both binding properties is achieved for more than one-third of the antibodies (37%; Fig. 4D). We also observed strong tradeoffs between affinity and non-specific binding, as most antibodies (87%) with high affinity (greater than wild type) also displayed relatively high non-specific binding (>50% of wild type). Conversely, most antibodies (91%) with large reductions in non-specific binding (<50% of wild type levels) displayed reduced affinity (less than wild type). This demonstrates the strong intrinsic tradeoffs between antibody affinity and specificity for variants with heavy chain CDR mutations that include sites strongly involved in both on- and off-target interactions.

### Prediction of novel mutations that further co-optimize antibodies

We identified a lead candidate (EM1) for further optimization that displayed an attractive combination of increased antigen binding (1.20x of wild type) and reduced non-specific binding (0.51x of wild type; Fig. 4D). We also selected additional clones for further mutagenesis, although to a more limited extent, to investigate the potential for optimizing antibody mutants with a diverse range of properties. We sought to predict novel CDR mutations, including at previously non-mutated CDR sites, to improve both the affinity and specificity of EM1 and related variants beyond the Pareto frontier identified for the first-generation library. This is not possible using the OneHot models because they are defined based on the sequences of the antibody mutants in the library and subsequent models would be insensitive to variations at novel sites in the CDRs. However, we reasoned that it may be possible to predict beneficial novel mutations using models that incorporate other types of molecular features that are based on the entire V_H_ domain.

Therefore, we evaluated two additional sets of molecular features based on antibody V_H_ domains for integration into models that predict the impacts of novel mutations on antibody affinity and specificity. The first set of features are Unified Representation (UniRep) features, which are deep learning features obtained from a neural network trained on over twenty million unlabeled protein sequences to perform next amino acid prediction^35^. A compelling aspect of these features is that they are not biased by assumptions regarding which molecular features are most important for antibody affinity and specificity. We used the previously reported 64-unit neural network to generate 64 UniRep features per antibody^35^. The second set of features, which we refer to as PhysChem features, are 26 physicochemical features that are based on the V_H_ domain sequence, including the isoelectric point, average residue hydrophobicity, and number of specific amino acids.

We next used the UniRep and PhysChem features to build LDA models for predicting both antibody affinity and specificity metrics (Supplementary Fig. 7), as we did for the OneHot features (Fig. 3A, B), and first evaluated their ability to classify the emibetuzumab mutants in the deep sequencing datasets. Encouragingly, we observed strong accuracies for classifying antibody affinity, with modestly higher accuracy for the UniRep model (91%) relative to the PhysChem model (85%; Supplementary Fig. 7). Likewise, we also observed strong accuracies for classifying antibody specificity (92% for both models). These classification accuracies were similar to those obtained for k-nearest neighbors models using the same features (80–86% for affinity and 88–91% for specificity; Supplementary Fig. 8). We also tested whether LDA projections trained on UniRep or PhysChem features correlated with continuous measures of affinity and specificity (Supplementary Fig. 7), as we did for the OneHot model (Fig. 3C, D). Encouragingly, our results were highly similar for the three sets of features, as model predictions were strongly correlated with experimental measurements of affinity and specificity.

We also evaluated the ability of the UniRep and PhysChem LDA models to predict property metrics of soluble IgGs, as we did for the OneHot LDA models (Fig. 4). We observed similar (albeit modestly lower) Spearman correlation coefficients between model predictions and experimental measures of affinity (0.66 for UniRep and 0.68 for PhysChem relative to 0.72 for OneHot) and specificity (0.65 for UniRep and 0.74 for PhysChem relative to 0.77 for OneHot; Supplementary Fig. 7). We also repeated this analysis using neural network models and observed similar performance relative to the OneHot models for affinity (0.75 for UniRep relative and 0.68 for PhysChem relative to 0.72 for OneHot) and specificity (0.70 for UniRep and 0.71 for PhysChem relative to 0.77 for OneHot; Supplementary Fig. 9). Overall, these results demonstrate that both simple (LDA) and more complex (neural net) models are capable of predicting continuous metrics that are strongly correlated with antibody properties based on both simple (OneHot) feature sets that are limited to the observed mutations in the sequenced library and more complex (PhysChem and UniRep) feature sets that can be potentially used to predict novel mutations features.

As a preliminary test of the generality of the UniRep and PhysChem LDA models, we evaluated the ability of the models to predict the impacts of CDR mutations on antibody affinity and specificity for residues that were selectively omitted during the training process, which we refer to as a leave-one-out analysis^45^. We performed such analysis by training the models on new sets of antibodies (4,000 sequences) that lack information about a given wild-type residue at one of the eight mutated CDR sites (i.e., no antibodies with the wild-type residue at a given CDR site were included in the training process). Next, the trained models were tested on antibodies that have the corresponding wild-type CDR residue regardless of the identity of the residues at the other seven mutated CDR sites (Supplementary Fig. 10). While the UniRep and PhysChem models both performed reasonably well, the UniRep models displayed the best overall performance. We repeated this process by training LDA models that lack information about the most common residue at each CDR site that was not wild type and observed that the UniRep models again displayed the best overall performance.

**Fig. 5:**
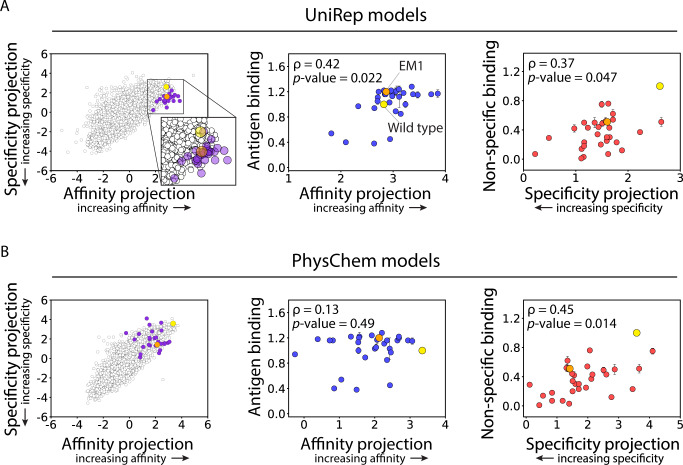
Models trained with deep learning features generalize to novel mutational space. Models trained using (**A**) deep learning (UniRep) and (**B**) physicochemical (PhysChem) features were used to evaluate the Pareto frontier. (**A**, left panel) A panel of 29 antibody variants with novel CDR mutations (not sampled in the original library) were designed along and beyond the Pareto frontier using the model trained with UniRep features. The predictions were limited to evolutionary conserved mutations with Blosum62 scores ≥0. (**B**, left panel) The designed antibody mutants using the UniRep models led to different distributions relative to the Pareto frontier predicted using the PhysChem models. (**A**, middle and right panels) Comparison of UniRep model predictions and experimental measurements of antigen (middle panel, HGFR, 1 nM) and non-specific (right panel, ovalbumin, 0.1 mg/mL) binding for soluble IgGs. (**B**, middle and right panels) Comparison of PhysChem model predictions and experimental measurements of antigen (middle panel, HGFR, 1 nM) and non-specific (right panel, ovalbumin, 0.1 mg/mL) binding for soluble IgGs. In (**A**) and (**B**), the experimental measurements are averages of three independent experiments, and the error bars are standard deviations. Independent two-sided *t*-tests were performed to determine significance.

Encouraged by these results, we next directly tested if our models could generalize to novel mutational space (Fig. 5). Therefore, we predicted single mutations (29 unique variants) that had not been sampled in the original library, including mutations at 15 novel CDR sites in HCDR2 and HCDR3. These mutations were primarily predicted for the EM1 clone (20 of 29 variants), but also for seven additional clones with the goal of generating a wide range of predictions and experimental measurements. We reasoned that focusing on evolutionarily conserved mutations would be most productive, and only considered predicted mutations with relatively high conservation (Blosum62 substitution scores ≥ 0; see Methods for more detail)^46^. The antibody mutants were selected based on the UniRep predictions and, notably, some of them were predicted to exceed the Pareto frontier of the first-generation library (Fig. 5A, inset of left panel). Moreover, the predictions sample a range of increases in predicted affinity or specificity or both. The PhysChem models predict different affinity and specificity metrics for the same mutants, which led to different distributions of each property (Fig. 5B, left panel).

### Experimental validation of novel mutation predictions

To test these predictions, we generated the 29 antibody variants as soluble IgGs and evaluated their relative levels of affinity and non-specific binding (Fig. 5). Encouragingly, we observed significant correlations between the UniRep LDA predictions and both antibody affinity (Spearman’s ρ of 0.46 and *p* value of 0.01) and specificity (ρ of 0.43, *p* value of 0.02 and Pearson correlation coefficient (*r*) of 0.34; Fig. 5A). Conversely, we only observed significant correlations between PhysChem LDA predictions and antibody specificity (ρ of 0.45, *p* value of 0.01 and *r* of 0.49; Fig. 5B). Notably, the neural network models for UniRep and PhysChem features each only achieved statistical significance for one antibody property (Supplementary Fig. 11). Likewise, the OneHot LDA models, which only account for the absence of residues at previously mutated CDR sites, also only achieved statistical significance for one antibody property (*p* value of 0.01 for affinity and 0.27 for specificity). Finally, if we did not limit our predicted mutations to those with relatively high conservation (Blosum62 substitution scores ≥ 0) for our best models (LDA UniRep), statistically significant predictions were only observed for one of the antibody properties (*p* value of 0.03 for specificity and *p* value of 0.41 for antigen binding). Overall, these findings demonstrate that LDA models trained with deep learning features were superior at generalizing to novel mutational space relative to those trained with conventional physicochemical antibody features, which is consistent with the findings from the leave-one-out analysis (Supplementary Fig. 10). More generally, these findings demonstrate the great potential for using these approaches to predict antibody mutations at novel CDR sites that co-optimize multiple properties linked to therapeutic antibody performance.

**Fig. 6:**
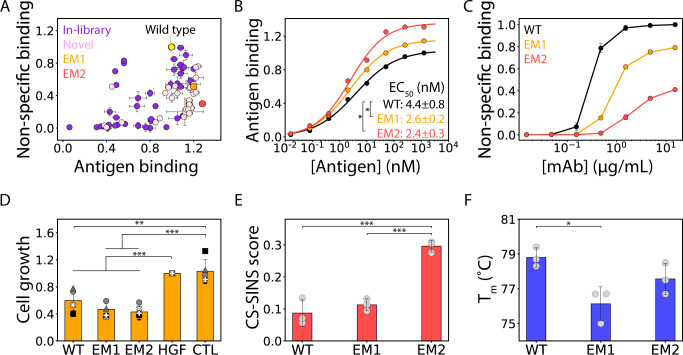
Emibetuzumab mutants co-optimized for affinity and specificity also display high bioactivity and stability. **A** Antigen (HGFR, 1 nM) and non-specific (ovalbumin, 0.1 mg/mL) binding measurements for soluble IgGs. **B** Concentration-dependent antigen binding of antibody mutants (EM1 and EM2) relative to wild-type. **C** Non-specific binding analysis of wild-type and mutant IgGs to a second polyspecificity reagent (soluble membrane proteins, 0.1 mg/mL). **D** Antibody-mediated inhibition of human cancer cell growth in response to hepatocyte growth factor (HGF). **E** Antibody self-association measurements (CS-SINS scores) in a standard formulation condition (pH 6 and 10 mM histidine). **F** Apparent melting temperature (*T*_*m*_) of the antibody mutants. In (**A**), the data are averages of three independent experiments and the error bars are standard deviations. In (**B**), the binding experiments were performed two or three times, representative binding curves are shown, the EC_50_ values are averages of the independent experiments, and the EC_50_ errors are standard deviations. The *p* value comparing WT to EM1 is 0.026 and the *p* value comparing WT to EM2 is 0.022. In (**C**), the binding experiments were performed three times and the average curve is shown. In (**D**), five independent experiments were performed (three technical replicates per experiment), the average value of each independent experiment is shown as a different symbol, the overall average values are shown as bars, the errors are standard deviations, and CTL is a control IgG (elotuzumab) that does not bind HGFR. The *p* values comparing WT, EM1, and EM2 to HGF are 4 × 10^−4^, 2 × 10^−6^, and 2 × 10^−7^, respectively. The *p* values comparing WT, EM1, and EM2 to CTL are 3 × 10^−3^, 2 × 10^−4^, and 2 × 10^−4^, respectively. In (**E**), the values are averages of three independent experiments, the error bars are standard deviations, and CS-SINS scores less than 0.35 correspond to IgG1s predicted to display low viscosity (<30 cP) and opalescence (<12 NTU) when concentrated to 150 mg/mL^13,44^. The *p* value comparing WT to EM2 is 0.0009 and the *p* value comparing EM1 to EM2 is 0.0002. In (**F**), the data are averages of three independent experiments and the error bars are standard deviations. The *p* value comparing WT to EM1 is 0.015. In (**B**) and (**D**–**F**), independent two-sided *t*-tests were performed to determine significance and the *p* values are <0.05 (*), <0.01 (**) and <0.001 (***).

We next plotted the experimental measurements of the relative antibody affinity and non-specific binding against each other for the 70 IgGs produced in this study, including the 29 IgGs with novel mutations absent in the original library, to identify the variants with the most co-optimal combinations of affinity and specificity (Fig. 6A). One variant (EM2) displayed particularly attractive properties, as it displayed increased antigen binding (1.28x) and reduced non-specific binding (0.30x) relative to wild type. Therefore, we evaluated this variant and its first-generation parental variant (EM1) in more detail. Affinity analysis of IgG binding as a function of antigen concentration revealed that both EM1 (EC_50_ of 2.6 ± 0.2 nM) and EM2 (EC_50_ of 2.4 ± 0.3 nM) have higher affinity than wild type (EC_50_ of 4.4 ± 0.8 nM; Fig. 6B). Despite the increased affinity of these variants, they both showed reduced non-specific binding to a second polyspecificity reagent (soluble membrane proteins) relative to wild type (Fig. 6C), which is consistent with similar non-specific binding measurements obtained using ovalbumin (Fig. 6A). Moreover, both EM1 and EM2 are at least as active at inhibiting hepatocyte growth factor-induced proliferation of human cancer cells as the wild-type antibody (Fig. 6D). EM1 and EM2 also have attractive, drug-like biophysical properties, as they display low levels of self-association (CS-SINS scores <0.35) in a standard antibody formulation (pH 6 and 10 mM histidine), which suggests the antibody mutants will display low viscosity and opalescence in concentrated antibody formulations (150 mg/mL; Fig. 6E)^47^. Moreover, these antibody variants display high thermal stability with melting temperatures >75 °C (Fig. 6F)^15^. Collectively, these results demonstrate the great potential of using machine learning for co-optimizing therapeutic antibodies to improve both affinity and specificity while maintaining high bioactivity and other drug-like biophysical properties.

**Fig. 7:**
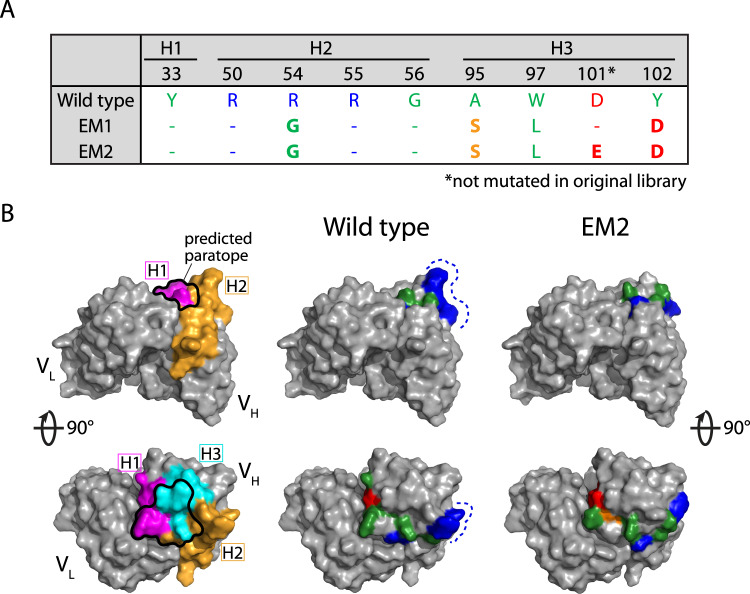
Structural modeling analysis of emibetuzumab mutants with co-optimized affinity and specificity. **A** Summary of the mutated CDR sites in the three heavy chain CDRs (H1, H2, and H3) for wild-type emibetuzumab and two mutants (EM1 and EM2). **B** Structural models of wild-type emibetuzumab and EM2 Fv regions. The predicted paratope is located primarily in the H1 and H3 CDRs and overlaps with the mutated sites. The structural models reveal a protruding positively charged patch (blue) in the wild-type antibody, which is located outside the predicted paratope, that is eliminated in the EM2 mutant. Some alterations to the paratope region and adjacent regions are also apparent in the EM2 mutant. The amino acids at the mutated sites in the structural models are colored as blue (Arg), red (Asp and Glu), green (Gly, Ala, Leu, Tyr, Trp) and orange (Ser).

Finally, we sought to understand the molecular basis for the improved antibody affinity and specificity of the best emibetuzumab variant (EM2; Fig. 7). This variant contained five CDR mutations relative to wild type, including one in HCDR2 and four in HCDR3 (Fig. 7A). Given the importance of both HCDRs on antibody affinity, we assumed that these mutations may be located at CDR sites that are outside of the paratope. However, we found that two of the five mutations occurred within the predicted paratope and there was some rearrangement within this region (Fig. 7B), which may be linked to the increased affinity of EM2 (Fig. 6B)^48^. The novel HCDR3 mutation in EM2 (D101E), which increases both affinity and specificity, is a conservative mutation located just outside the predicted paratope. Additional CDR mutations located outside the predicted antibody paratope, such as R54G in HCDR2, led to the removal of a large positively charged patch, which may explain the reduced non-specific binding given the strong link between positively charged patches in antibody CDRs and non-specific binding^24,49–52^. Overall, these findings suggest the combination of CDR mutations in EM2 that co-optimize affinity and specificity do so by largely preserving the paratope for high affinity binding while disrupting a positively charged patch outside the paratope to reduce non-specific binding.

## Discussion

We have developed a facile approach for the multi-objective optimization of therapeutic antibodies. We show that one-dimensional projections obtained from LDA models reflect the continuous variability of the evaluated biophysical properties. These projections, linear combinations of learned scalings that maximize interclass separation, are trained on binary datasets for affinity and specificity, yet are correlated with continuous property values, which enables the direct identification of Pareto optimal antibodies. Examination of the projection weights for OneHot features revealed that LDA scalings were strongly correlated with the site-specific enrichment of mutations in antibody libraries screened for high levels of antigen and non-specific binding (Supplementary Fig. 12). While we are unaware of previous work on predicting continuous metrics correlated with antibody properties such as affinity or non-specific binding from binary deep sequencing datasets, it is notable that this general approach has been pursued in unrelated fields of study. For example, in the field of text analysis, it has been demonstrated that continuum information can be predicted based on binary labels^53^. This approach used the outputs of a variety of trained binary classifiers to predict continuous sentiment scores associated with text samples. We believe that this general approach holds great potential for improving and simplifying antibody engineering.

Another aspect of our work that deserves further consideration is the demonstration of strict tradeoffs between antibody affinity and specificity at the library scale (thousands of mutants). Previous studies have elucidated similar tradeoffs for pairs of antibody and protein properties, including affinity/thermostability, affinity/solubility and humanness/thermostability^16,21,54–60^. However, these studies are generally limited by low-throughput measurements of relatively small sets of protein mutants (tens to hundreds of individually produced and experimentally measured variants)^16,21,54^. In contrast, our approach applies to much larger numbers of antibody mutants, the size of which is only limited by the deep sequencing technology. Moreover, previous studies that simultaneously optimized combinations of antibody properties such as humanness (e.g., human string content)^55^ and stability (e.g., AMBER energy calculations)^56^ are either not applicable for predicting other antibody properties given the lack of models (e.g., non-specific binding) or the difficulty in applying them due to the need for extremely accurate antibody/antigen structures (e.g., affinity). In contrast, our methods enable the simple identification of Pareto optimal clones directly from binary deep sequencing data without the need for pre-existing models, including those that require accurate antibody/antigen structural information. Our approach also enables the selection of specific levels of multiple antibody properties at the same time, affording greater control over the antibody engineering process than has been possible previously.

Another key aspect of our work is the ability to identify beneficial mutations that were absent in the original antibody library, enabling extrapolation to novel mutational space. A common goal of machine learning in the field of protein engineering is to develop generalizable algorithms that can extrapolate to unseen mutations, especially at non-mutated sites in the training sets. A recent study demonstrates the great potential of algorithms that use deep learning features to generalize to novel mutational space for improving the activity of enzymes and fluorescent proteins^36^.

Our models, which used similar types of deep learning features and generalize to novel mutational space, deserve further consideration. First, we found that it was necessary to limit the predictions to mutations with moderate-to-high evolutionary conservation (Blosum62 substitution scores ≥ 0). This was necessary because the models also predicted mutations with lower evolutionary conservation, such as tryptophan mutations in the CDRs, which we found resulted in levels of affinity and non-specific binding that were poorly correlated with the model predictions. We expect that additional information about the site-specific frequencies of mutations in antibody repertoires, which were not considered in this work, could be incorporated into future models to further improve predictions. Second, the deep learning features used in this work were generated from algorithms trained on millions of diverse proteins, including mostly non-antibody proteins^35,36^. We expect that similar deep learning features extracted from more closely related protein sequences, such as human antibody repertoires, will lead to even better model performance. Third, we used simple comprehensive scans of single mutations to predict novel sequences. This approach will not scale to the exponentially large antibody sequence space. However, the neural networks used here could serve as a starting point to implement generative neural networks that could enable the prediction of antibody sequences with defined affinities and specificities^61,62^. Fourth, the models in this work were trained to generalize to novel mutational space for predicting metrics correlated with two antibody properties (affinity and specificity) that could be readily screened in a high-throughput manner using massive antibody libraries. We expect that our approaches could be easily extended to other antibody properties that can be screened via in vitro library sorting. For example, our approach could be used for simplifying the selection of species cross-reactive antibodies. The lack of species cross-reactivity is a common problem in pre-clinical development because antibodies that bind their target human antigens with high affinity often weakly bind or fail to bind antigen orthologs from different species (e.g., mouse, rat and monkey) that are necessary for pre-clinical testing^63,64^. A key next step is to rigorously test the generality of our approaches for predicting the impacts of additional sets of CDR mutations in emibetuzumab as well as CDR mutations in other antibodies with unique physicochemical properties. We believe that these and other exciting opportunities are expected to lead to machine learning models that can increasingly generalize to novel mutational space and reduce the amount of required experimentation to obtain co-optimized drug-like antibodies.

## Methods

### Antibody sub-library design and generation

Sites in the heavy chain CDRs of emibetuzumab were selected for mutagenesis using chemical rules reported previously for predicting antibodies with drug-like specificity^38^. Briefly, CDR sites in the V_H_ domain were selected for mutagenesis if they were (i) flagged by one or more of the six maximum chemical rules, (ii) hydrophobic or positively charged, (iii) solvent exposed (>10%), and (iv) relatively uncommon in human antibodies (<50% site-specific frequency in human repertoires)^65,66^. For each of eight sites that were identified in the heavy chain CDRs for mutagenesis (Y33, R50, R54, R55, G56, A95, W97, and Y102), degenerate codons were selected to sample the wild-type residue in addition to five additional residues which sample a range of physicochemical properties and were predicted to reduce non-specific binding (Fig. S1). The final library of yeast-displayed single-chain Fabs (scFabs; theoretical diversity of 1.7 × 10^6^) was constructed via homologous recombination following electroporation of EBY100 *Saccharomyces cerevisiae*^67^.

### Preparation of antigen and polyspecificity reagents

For MACS selections, Protein A Dynabeads (Invitrogen, 10002D) were coated with the extracellular domain of hepatocyte growth factor receptor as an Fc fusion protein (HGFR-Fc; Acro Biosciences MET-H5256). For FACS selections, HGFR-Fc was used as purchased following reconstitution. Soluble membrane proteins (SMPs) were prepared as previously described^39,40^, and SMPs and ovalbumin (OVA; Sigma A5503) were biotinylated with Sulfo-NHS-LC-Biotin (Pierce, P121335).

### Antibody library sorting and deep sequencing

The first two rounds of MACS were performed by incubating 10^7^ HGFR-Fc coated Dynabeads with 10^9^ (Round 1) and 10^7^ (Round 2) yeast cells (displaying scFabs) for 3 h at room temperature in PBSB with 1% milk. Binding cells were isolated using a magnet and regrown in selective media. Further sorting was performed with a FACS sorter following incubation of 10^7^ regrown yeast cells with soluble reagents. The antigen (1 nM HGFR-Fc with 1% milk in PBSB) and ovalbumin (260 μg/mL in PBSB) were incubated with yeast cells for 3 h at room temperature. Soluble membrane proteins (130 μg/mL in PBSB) were incubated with yeast cells for 20 min on ice. A mouse anti-Myc mAb (1:1000; Cell Signaling Technologies, 2276 S) was co-incubated with the antigen and polyspecificity reagents to evaluate antibody display. The bound reagents were detected using different secondary reagents. The biotinylated polyspecificity reagents were detected using streptavidin AF647 (1:1000; Life Technologies, S32357). The antigen (HGFR-Fc) was detected with goat anti-human AF647 (1:300; Jackson ImmunoResearch Labs, 109605098). The anti-Myc tag antibody was detected using goat anti-mouse AF488 (1:300; Life Technologies, A11001).

Cells were sorted on a MoFlo Astrios sorter (Beckman-Coulter) by selecting those that expressed scFabs (as detected using the anti-Myc tag antibody) and displayed high antigen binding or high and low polyspecificity reagent binding (Supplementary Fig. 2). Gates for antigen sorting were set to collect the top 50% of antigen-positive cells, capturing those along the top of the diagonal population of antigen-binding and antibody-displaying cells. Gates for specificity (non-specific binding) sorting were set to capture the top 25% of binders (also along the top of the diagonal population of binding and displaying cells) and the bottom 10% of binders. The selected populations of cells in round 3, which include high antigen binding and high and low non-specific binding to two polyspecificity reagents, along with the input library, were deep sequenced. Given that the sorting experiments were performed in duplicate, this resulted in a total of 12 library samples that were deep sequenced. The deep sequencing was performed as reported previously^38^.

In total, sequencing covered approximately 20% of the total library diversity with 1,504,269 unique sequences acquired in the first replicate (6 samples: input, antigen positive, PSR positive, PSR negative, ovalbumin positive, ovalbumin negative) and 851,151 unique sequences in the second replicate (6 samples). The reproducibility between replicates is shown in Supplementary Fig. 13.

The deep sequencing was processed using the following procedure. First, a positive label for antigen binding was assigned to sequences found in both antigen positive samples, and a negative label was assigned to those not found in either sample but were found in the input library sample. Sequences found in only one antigen positive sample were discarded. A positive label for non-specific binding was assigned to sequences found in at least three of the four positive samples and none of the negative samples for non-specific binding to soluble membrane proteins and ovalbumin and a negative label was assigned to sequences found in at least three out of the four negative samples and none of the positive samples for non-specific binding. Unlabeled antibody sequences, as well as sequences with gaps or unidentified residues, were discarded. Next, 4000 of the most frequently observed antibody sequences, prioritized for positive antigen binding labels due to their lower abundance and stratified evenly in terms of high and low specificity, were identified and used as the master sequence dataset for machine learning analyses. The final dataset consisted of 2000 high specificity and 2000 low specificity clones, with 1516 of these displaying high antigen binding and 2484 displaying low antigen binding. All sequences were 115 residues long, ranging from H1 to H113 (Kabat numbering). Before final model development, we confirmed that our sample size was sufficient to achieve maximum performance (Supplementary Fig. 14). Less than 1% of sequences had mutations at sites that were not intentionally mutated in the library. This dataset is reported in Supplemental file emi_binding.csv.

For the conventional analysis of the deep sequencing data, frequency values were calculated using the same criteria as used for sample observation (as reported above) to maintain consistency. Frequency was calculated by dividing the number of occurrences in each sample by the number of samples successfully sequenced. For the analyzed individual clones (scFabs and IgGs), sequences were considered in the subsequent analysis if they were observed in at least three of the four negative non-specific binding samples (ovalbumin and soluble membrane proteins) and both antigen positive binding samples. Enrichment ratios for antibody sequences were calculated as the log_2_ of the ratio of output to input frequencies for each replicate. Final frequency values and enrichment ratios were calculated as averages of the two deep sequencing replicates.

### Antigen binding analysis using yeast surface display

Individual clones were isolated and Sanger sequenced from the enriched libraries (after FACS sorting) to generate a reference set of measurements of antibody affinity and specificity for model evaluation. The on- and off-target binding properties of the scFabs (displayed on yeast) were evaluated using a BioRad Zeti5 flow cytometer. Briefly, single-point binding of HGFR-Fc or OVA to scFabs on yeast (10^6^ cells for HGFR-Fc and 5 × 10^6^ cells for OVA) was performed and detected as described above. Only cells that expressed scFabs were considered in subsequent quantitative analysis. Single-point binding measurements are reported as the mean binding signal divided by the mean display signal, both of which were gated for antibody expression. The signal for the scFab display-only control (no HGFR-Fc or OVA added) was subtracted from the binding signals for each sample (with HGFR-Fc or OVA). The resulting binding measurements were normalized to the binding measurements for the wild-type emibetuzumab scFab and represent averages of two to three biological replicates.

### Production of soluble IgGs

Variable heavy (V_H_) domains of selected in-library clones were isolated from yeast display plasmids or ordered as geneblocks (IDT). The V_H_ domains were cloned into pTT5 mammalian expression plasmids containing a common IgG1 heavy and light chain (kappa) framework, as described previously^39^. Briefly, the PCR-amplified fragments and expression vectors were digested with the desired restriction enzymes (EcoRI-HF and NheI-HF for V_H_; EcoRI-HF and BsiWI-HF for V_L_; New England Biolabs). Finally, digested DNA fragments and vectors were purified (Qiagen, 28104), ligated with T4 ligase (New England Biolabs, M0202L) and transformed into competent DH5α cells. Antibody sequences were confirmed by Sanger sequencing.

Single mutations were introduced into the V_H_ domain sequences through site-directed mutagenesis using primers that amplified the entire plasmids. High-fidelity polymerase (PfuUltra II Hotstart PCR Master Mix, Agilent 600850) was used to avoid undesired mutations. Following PCR, the product was digested with DpnI (NEB R0176S) for 30 min to remove the parent template. Finally, plasmids were isolated and sequenced to screen for the desired mutations after transformation.

For IgG expression, the HEK293-6E cell line (L-11565, National Research Council Canada) was cultured in disposable conical tubes (Corning, 7203954, Thermo Fisher Scientific) with F17 (50591354, Thermo Fisher Scientific) or BalanCD HEK293 (91165, Fujifilm Irvine Scientific) media. The cultures were incubated at 37 °C and 250 rpm. Soluble IgGs were produced via transient transfection (30 mL) using 7.5 μg each of heavy and light chain plasmids, and 60 μg polyethylenimine (PEI MAX, 247651, Polysciences Inc.) for F17 media or 75 μg polyethylenimine for BalanCD media. Five days after transfection, cultures were harvested and the supernatant was batch purified using Protein A agarose resin (20334, Pierce; Thermo Fisher Scientific) followed by size-exclusion chromatography (SEC) using a Shimadzu Prominence semi-prep HPLC System outfitted with a LC-20AT pump, SIL-20AC autosampler, and FRC-10A fraction collector. Proteins were loaded onto an SEC column (Superdex 200 Increase 10/300 GL column; GE, 28990944) and analyzed at 0.75 mL/min using a PBS running buffer with 200 mM arginine (pH 7.4) After purification, soluble IgGs were buffer exchanged into PBS (pH 7.4) with Zeba desalting columns (89890, Thermo Fisher Scientific), aliquoted, snap-frozen, and stored at −80 °C.

### Antibody affinity and non-specific binding measurements

Affinity analysis was performed as reported previously^39^. Briefly, Protein A Dynabeads (Invitrogen, 10002D) were washed three times and diluted to 54 μg/mL in PBSB. Beads (30 μL) were incubated with antibodies (85 μL, 15 μg/mL) overnight at 4 °C. The coated beads were then washed twice by centrifugation (3500x g for 4 min) with PBSB. For single-point binding measurements, biotinylated reagents [1 nM biotinylated-HGFR (Sino Biological 10692-H27H-B), 0.1 mg/mL ovalbumin, 0.1 mg/mL SMP] in PBSB were incubated with the washed beads. Biotinylated SMP was incubated at 4 °C for 20 min, as previously reported^2,3^. Biotinylated HGFR and OVA were incubated for 3 h at room temperature. The beads were then washed once and incubated with streptavidin-AF647 (1:1000; Invitrogen, S32357) and goat anti-human Fc F(ab’)_2_ AF-488 (1:1000; Invitrogen, H10120) on ice for 4 min. Finally, the beads were washed once more, resuspended in PBSB, and analyzed via flow cytometry to measure their median fluorescent intensities (MFI). Results are reported as normalized scores between emibetuzumab and elotuzumab as the high and low binding controls, respectively. Affinity and non-specific binding measurements are reported in iso_binding.csv and igg_binding.csv files.

EC_50_ values of antigen binding were measured for select variants as IgGs on beads. Washed Protein A beads were incubated with antibodies (85 μL, 0.076 μg/mL) overnight. The coated beads were washed with PBSB and incubated with biotinylated HGFR in PBSB at a range of concentrations at 10x molar excess for 3 h at room temperature. The beads were then washed once and incubated with secondary reagents (1:1000; streptavidin-AF647, 1:1000; goat anti-human Fc F(ab’)_2_ AF-488) on ice for 4 min. Finally, the beads were washed once more, resuspended in PBSB, and analyzed via flow cytometry to measure their median fluorescent intensities (MFI).

Dose-dependent non-specific binding was measured for select variants was measured as IgGs on beads. Washed Protein A beads were incubated with antibodies (85 μL) at a range of concentrations (0.015–15 μg/mL) overnight. The coated beads were washed with PBSB and incubated with biotinylated SMP and OVA in PBSB. The beads were then washed once and incubated with fluorescent secondary reagents on ice for 4 min. Finally, the beads were washed once more, resuspended in PBSB, and analyzed via flow cytometry to measure their median fluorescent intensities. Results are reported as normalized scores between emibetuzumab and elotuzumab at the highest antibody concentrations incubation (15 μg/mL) as the high and low binding controls, respectively.

### Cell proliferation

NCI H596 cells (ATCC, HTB-178) were seeded at 2,000 cell/well in 96 well plates (Fisher, 14387220) in RPMI1640 supplemented with 10% FBS (Fisher, 10082147) and antibiotics (penicillin-streptomycin; Fisher, 15-140-122). After growth overnight, cells were preincubated with antibodies (100 nM) for 30 min before stimulation with 50 ng/mL HGF (Fisher, 501624085) for five days. Promega CellTiter-Glo™ Luminescent Cell Viability Assay Kit (Fisher, PRG7572) was used to measure cell proliferation on a Biotek Synergy H1 Hybrid Microplate reader (Biotek) according to the manufacturer’s instructions.

### Charge-stabilized self-interaction nanoparticle spectroscopy (CS-SINS)

CS-SINS was measured as reported previously^47^. Briefly, capture antibody (Jackson ImmunoResearch, 109-005-008) and polylysine (90%:10% w/w ratio respectively; Fisher Scientific, ICN19454405) were immobilized on concentrated gold nanoparticles and incubated overnight. Dilute IgG solutions (11.1 μg/mL, 45 μL) were incubated with 5 μL of gold conjugates for 4 h at room temperature. Absorbance spectra was measured on a Biotek Synergy Neo plate reader (Biotek) in 1 nm increments between 450 and 650 nm. A quadratic equation was fit to describe the forty data points surrounding the maximum measured absorbance. The inflection point of this quadratic was calculated to determine the plasmon wavelength. Plasmon wavelengths were then normalized and calibrated against a panel of five antibodies (NIST mAb, ibalizumab, mepolizumab, trastuzumab, and romosozumab). This calibration panel was used to rescale the measurements to the same scale as reported in the original study^47^.

### Melting temperature analysis

Melting temperatures of soluble IgGs were analyzed via differential scanning fluorimetry. Antibodies (0.12 μg/mL) were combined with Protein Thermal Shift Dye (Applied Biosystems, 4461146) in a 7:1 antibody:dye molar ratio. Samples were submitted to the University of Michigan Advanced Genomics Core for analysis using an ABI Prism 7900HT Sequence Detection System (Applied Biosystems). Fluorescence was evaluated for 45 min at temperatures increasing from 25 to 98 °C. Background signals (buffer only samples) were subtracted from the results and melting temperatures were calculated as the turning point of the first unfolding event.

### Molecular feature embedding

(1) One-hot encoding was performed on V_H_ sequences using the scikit-learn (1.0.1) python package. Sequences were first encoded as integers using sklearn.LabelEncoder and subsequently one-hot encoded using sklearn.OneHotEncoder. Code to generate one-hot encoded features is provided as onehot_gen.py. (2) PhysChem features were extracted by calculating 26 biophysical descriptors that describe the entire V_H_ domain. This includes the count of all twenty amino acids as features 1–20, the number of hydrophobic (A, I, L, F, V), amphipathic (W, Y, M), polar (Q, N, S, T, C, M), and charged residues (K, R, D, E, H) as features 21–24, the isoelectric point using ExPASy as feature 25, and the average hydropathy score of all residues using Kyte-Doolittle scoring index (Table S1) as feature 26^68^. ExPASy calculations of isoelectric point are reported in emi_pI.txt, igg_pI.txt, and iso_pI.txt. Code to generate PhysChem features is included as physchemvh_gen.py. (3) UniRep features for V_H_ sequences were acquired using a previously reported procedure^69^. We used the base model weights for the UniRep-64 neural network. Briefly, a babbler64 object was instantiated and the built-in get_rep() function was used to compute UniRep features for each V_H_ sequence, which resulted in 64 UniRep features per antibody variant. UniRep features for each dataset are reported in emi_reps.csv, iso_reps.csv, and igg_reps.csv.

### Model development

Analyses were performed in python (3.8) using the scikit-learn (1.0.1) and TensorFlow (2.7.0) packages. For the LDA models, the built-in LinearDiscriminantAnalysis function with the singular value decomposition solver was used. Independent LDA models were trained to predict metrics correlated with antigen binding and polyspecificity reagent (ovalbumin and soluble membrane proteins) binding with five-fold cross-validation performed, consisting of 3200 sequences in each training set and 800 sequences in each test set. After confirming that training and test set accuracies were similar, the final models were trained on the full datasets. Finally, one-dimensional projections were generated for each sequence from the final trained models for both antigen and polyspecificity reagent (ovalbumin and soluble membrane proteins) binding. This process was repeated independently for each set of features (OneHot, PhysChem, and UniRep). Model code is included as onehot_models.py, physchemvh_models.py, and unirep_models.py. To predict metrics correlated with properties of previously unseen sequences, features for novel sequences were generated, and the trained LDA models were used to generate one-dimensional projections. These novel sequences sampled all single amino acid mutations except cysteine at all sites in HCDR2 and HCDR3. HCDR1 was excluded because the original dataset only contained one mutation in HCDR1. The features from each novel sequence were generated as described above. Antibody sequences at or near the Pareto frontier were identified that displayed a range of predicted affinity and specificity metrics. Evolutionarily conservative mutations were defined using the BLOSUM62 scoring matrix^70,71^. Baseline k-nearest neighbors models were created using the built-in KNeighborsClassifier function. Classifiers were trained using a range of nearest neighbor values from 1 to 25 and the mean test accuracy from five-fold cross-validation was reported.

The neural network models were built and trained in TensorFlow. The neural network architecture consists of a two-layer neural network that generates a one-dimensional projection, called the projector network, and a final prediction layer that predicts accuracy or specificity based on the projection (Supplementary Fig. 5). The neural networks were trained for predicting metrics correlated with affinity and specificity for all three feature sets, which led to the generation of a total of six neural networks. Each network was trained using the SparseCategoricalCrossentropy loss function, ADAM optimizer, a batch size of 50 sequences, and a total of 3200 sequences for training. Five-fold cross-validation accuracy analysis was used to identify the number of antibody sequences resulting in plateauing of model training accuracy, and subsequently final models were trained for 50 epochs (OneHot and PhysChem features) or 250 epochs (UniRep features). The networks were trained using the same data used to train the LDA models.

### Antibody structural modeling

Molecular Operating Environment (MOE) software (2021.05) was used to generate homology models. Amber10:EHT forcefield and a dielectric constant of 4 were also used. Antibody modeler was implemented to identify template structures from a Fab/antibody structure database for V_H_, V_L,_ and individual CDR loops. For emibetuzuab, the V_H_ and V_L_ chains of PDB:4LIQ were selected as the framework templates. PDB:3W2D was used for the light chain CDR loop templates, PBD:4YHL was used for the heavy chain CDR 1 and 2 templates, and PDB:5NHW was used for the heavy chain CDR3 template. Finally, the initial antibody model was energy minimized with a minimum gradient setting of 0.00001 RMS kcal/mol/Å^2^. The final homology models for the emibetuzumab variants were then exported to PyMOL (2.5) for visualization.

### Statistical analysis and visualization

All data (except homology models) were plotted and visualized using the python packages matplotlib (3.4.3) and seaborn (0.11.2). Statistical analysis was performed in python (3.8) using the scipy (1.7.3) package. The normality of the distributions of variables was evaluated using the Shapiro-Wilks test^72^. Using this test, most distributions of the experimental data and model predictions (except for the experimental non-specific binding data in Fig. 5) differed significantly from normal distributions (*p* < 0.05). For all datasets, the Spearman correlation coefficients are given. If both the experimental data and model predictions did not significantly differ from normal distributions, Pearson correlations are also reported. The statistical significance of Spearman and Pearson correlations were calculated by a two-sided student’s t-test.

### Reporting Summary

Further information on research design is available in the Nature Research Reporting Summary linked to this article.

## Supplementary information

Supplementary Information

Reporting Summary

## Data Availability

Source data are provided with the paper, online at BioProject (Accession code: PRJNA850089), and in the Tessier lab GitHub repository: https://github.com/Tessier-Lab-UMich/Emi_Pareto_Opt_ML.

## Source data

Source Data

## Code availability

Scripts to generate main figures and perform data analysis are provided in the Tessier lab GitHub repository: https://github.com/Tessier-Lab-UMich/Emi_Pareto_Opt_ML.
