# Supplementary material for: Co-optimization of therapeutic antibody affinity and specificity using machine learning models that generalize to novel mutational space
Source: Nat Commun. 2022 Jul 1;13:3788. doi: 10.1038/s41467-022-31457-3 (PMC9249733; doi:10.1038/s41467-022-31457-3)
Supplement: Supplementary file 1 — Supplementary Information [file 41467_2022_31457_MOESM1_ESM.pdf]

Supplementary Information

Co-optimization of therapeutic antibody affinity and specificity using machine learning models that generalize to novel mutational space

Emily K. Makowski, Patrick C. Kinnunen, Jie Huang, Lina Wu, Matthew D. Smith, Tiexin Wang, Alec A. Desai, Craig N. Streu, Yulei Zhang, Jennifer M. Zupancic, John S. Schardt, Jennifer J. Linderman, and Peter M. Tessier

Supplemental Table 1

Table S1. Hydropathy values used for the PhysChem model. The hydropathy values were reported previously (Kyte et al., *J. Mol. Biol*, 1982).[1]

Residue	Hydropathy score
A	1.8
C	2.5
D	-3.5
E	-3.5
F	2.8
G	-0.4
H	-3.2
I	4.5
K	-3.9
L	3.8
M	1.9
N	-3.5
P	-1.6
Q	-3.5
R	-4.5
S	-0.8
T	-0.7
V	4.2
W	-0.9
Y	-1.3

Supplemental Figures 1-14

A

Emibetuzumab																	
H1	26	27	28	29	30	31	32	33	34	35							
	**G**	**Y**	**T**	**F**	**T**	D	**Y**	**Y**	**M**	**H**							
H2	50	51	52	52A	53	54	55	56	57	58	59	60	61	62	63	64	65
	**R**	**V**	N	P	N	**R**	**R**	**G**	**T**	**T**	Y	N	**Q**	**K**	F	E	**G**
H3	93	94	95	96	97	98	101	102									
	**A**	**R**	**A**	N	**W**	**L**	D	**Y**									

B

	H1	H2				H3		
Position	33	50	54	55	56	95	97	102
Wild type	**Y**	**R**	**R**	**R**	**G**	**A**	**W**	**Y**
Sampled	F	K	K	K	A	F	L	F
	V	G	G	G	N	V	V	V
	A	A	A	A	S	Y	G	A
	S	T	T	T	T	S	A	S
	D	E	E	E	D	D	S	D
Codon	KHY	RVR	RVR	RVR	RVY	KHY	KBG	KHY

C

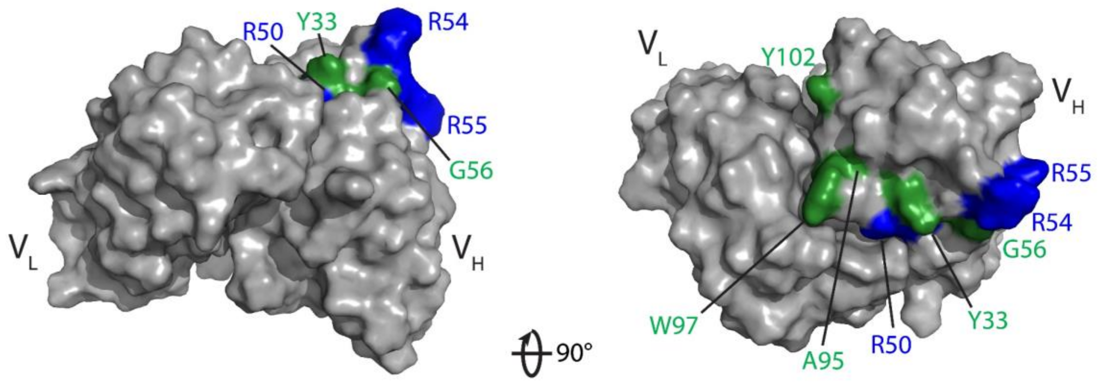

Figure S1. Design of an emibetuzumab V_H library. (A) Eight sites in the heavy chain CDRs (boxed with bold green or blue font) were selected for mutation based on predictions of sites involved in non-specific binding.[2] More specifically, the CDR sites were selected if they were i) flagged by the one or more of the six maximum chemical rules, ii) hydrophobic or positively charged, iii) solvent exposed (>10%) and iv) relatively uncommon in human antibodies (<50% frequency in human repertoires). For each of eight sites that were identified in the heavy chain CDRs (Y33, R50, R54, R55, G56, A95, W97, and Y102), degenerate codons were selected to sample the wild-type residue and five additional residues that sample a range of physicochemical properties and were predicted to reduce non-specific binding. The bolded black residues were flagged by a maximum chemical rule but did not meet other criteria. Residue K62 met the criteria but was not mutated. (B) Degenerate codons were used to create the antibody library, which resulted in sampling five mutations and the wild-type residue at each of the eight CDR sites. (C) Structural model of the emibetuzumab Fv region with the mutated sites highlighted.

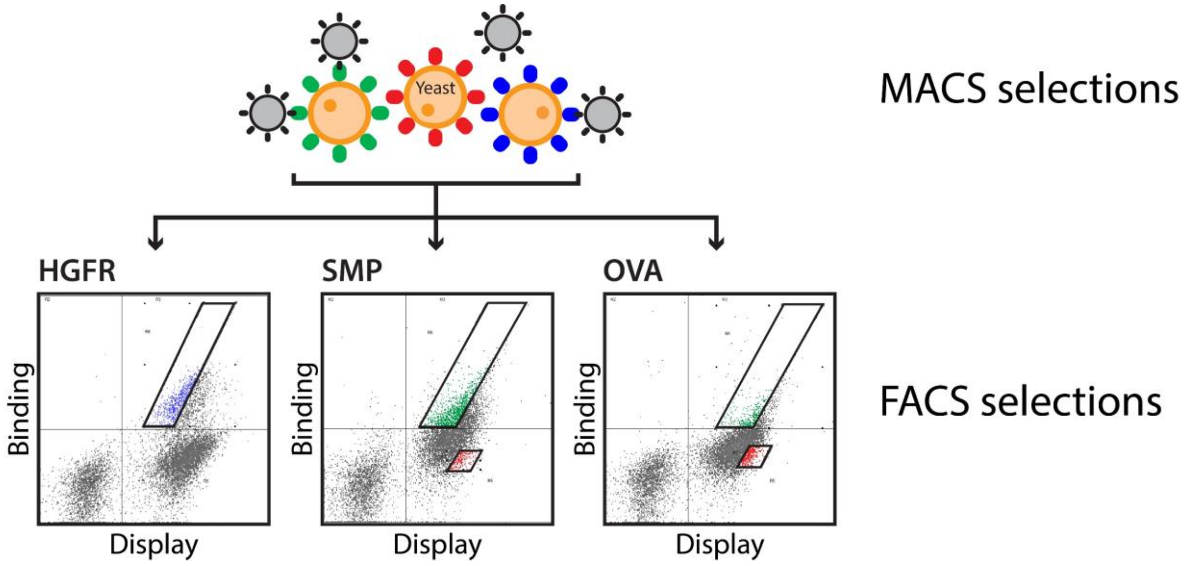

Figure S2. Library sorting strategy. Single-chain Fab libraries displayed on yeast were incubated with antigen (HGFR)-coated magnetic beads and sorted using MACS twice to remove non-binding variants. The library was then sorted using FACS for antigen binding, and binding and non-binding against soluble membrane proteins (SMP) and ovalbumin (OVA). The top 50% of the antigen-positive population (blue), the top 25% of the SMP and OVA populations (green), and the bottom 10% of the SMP and OVA populations (red) were collected and deep sequenced.

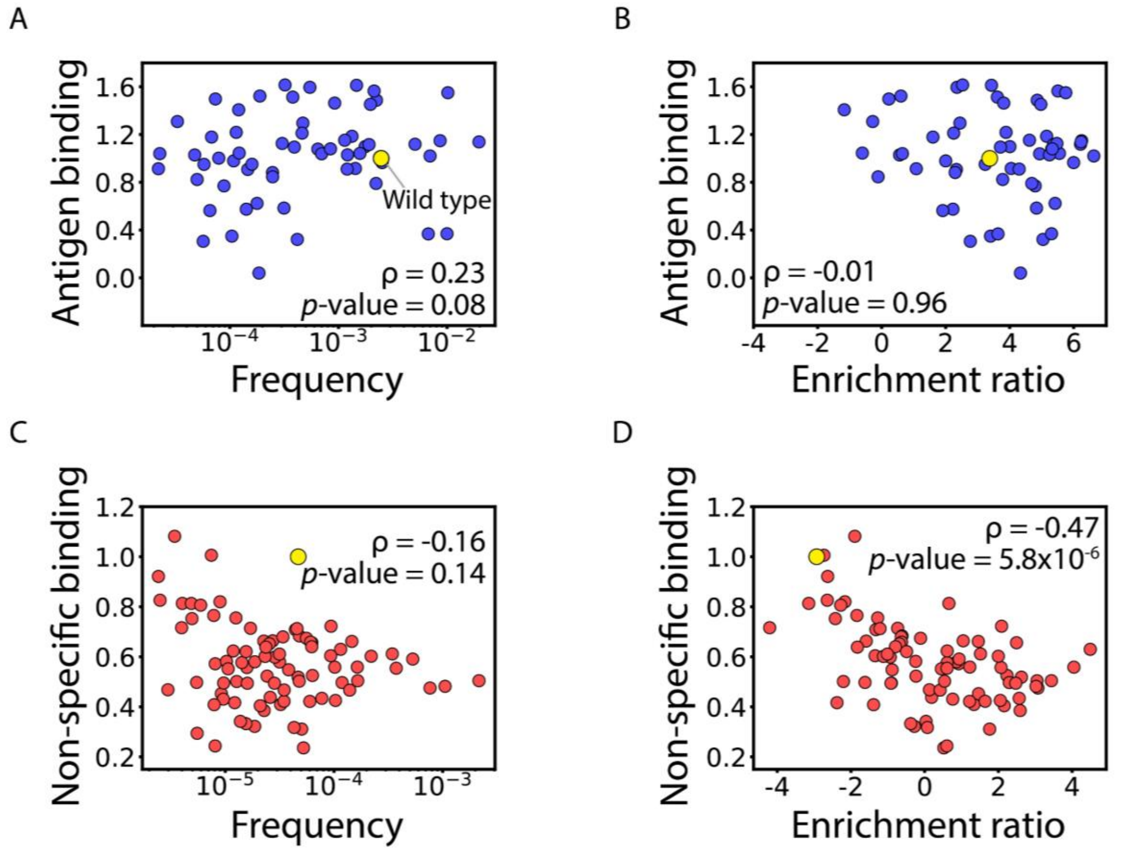

Figure S3. Conventional analysis of the deep sequencing data is generally poorly predictive of antibody mutants with either improved affinity or specificity. (A-D) The deep sequencing data sets for the libraries sorted for high affinity and low non-specific binding (ovalbumin) were used to evaluate the (A, C) frequencies and (B, D) enrichment ratios of each antibody mutant, and the values were correlated with the experimental measurements of antigen binding and non-specific binding collected as single-chain Fabs on yeast. The experimental measurements were performed three times and the average values are reported. Independent two-sided *t*-tests were performed to determine significance.

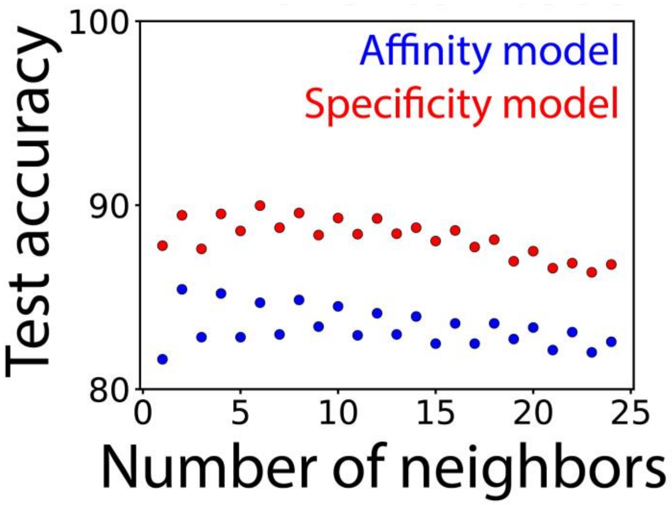

Figure S4. K-Nearest Neighbors (KNN) classification of antibody affinity and specificity. KNN models were trained for classifying antibody affinity and specificity (4000 antibodies) using OneHot features. Classification accuracies for a range of k values were evaluated using 5-fold cross validation, with average test accuracies shown. Overall model accuracies of 83% (affinity) and 89% (specificity) were achieved using a number of neighbors = 5.

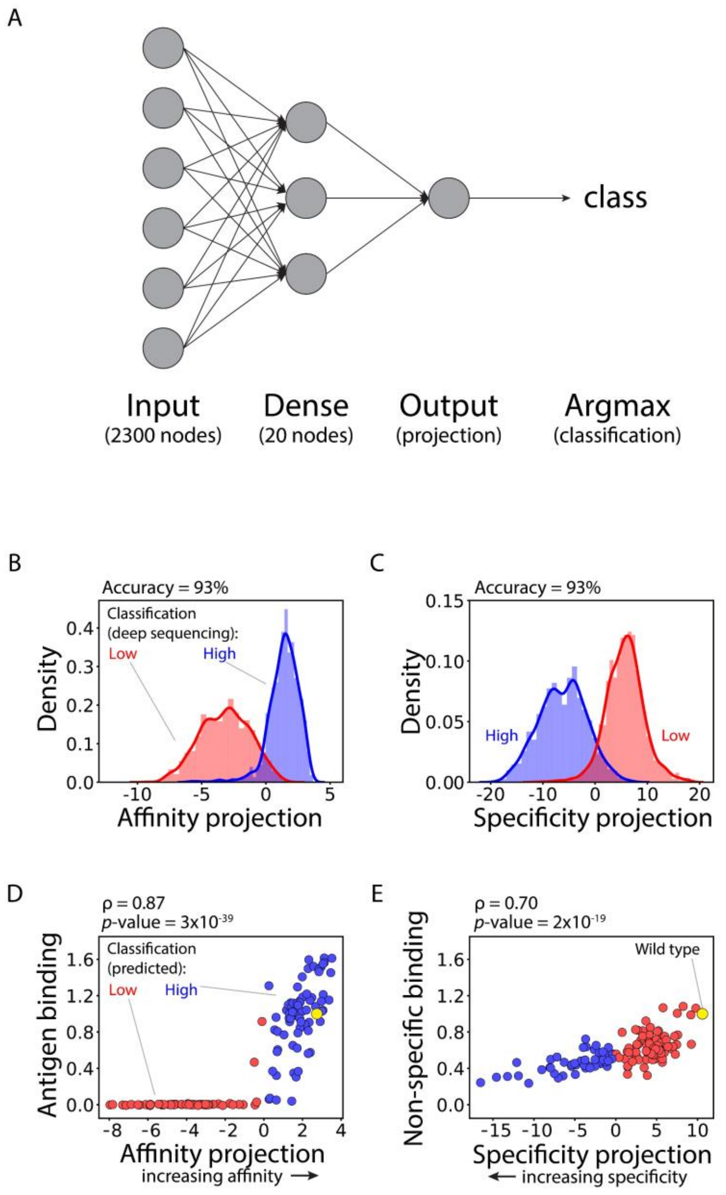

Figure S5. Neural network models enable continuous predictions of antibody affinity and specificity. (A) Schematic showing the simple neural network model structure used for comparison with the LDA models. The networks contain an intermediate layer of nodes and classify antibodies based on their projections to a single-value dense layer. (B-C) The neural network models were trained using OneHot features and displayed high accuracy for classification of both affinity and specificity. (D-E) The neural network model projections were strongly correlated with continuous measurements of antibody affinity and specificity. The experimental measurements are described in Figure 3. Independent two-sided *t*-tests were performed to determine significance.

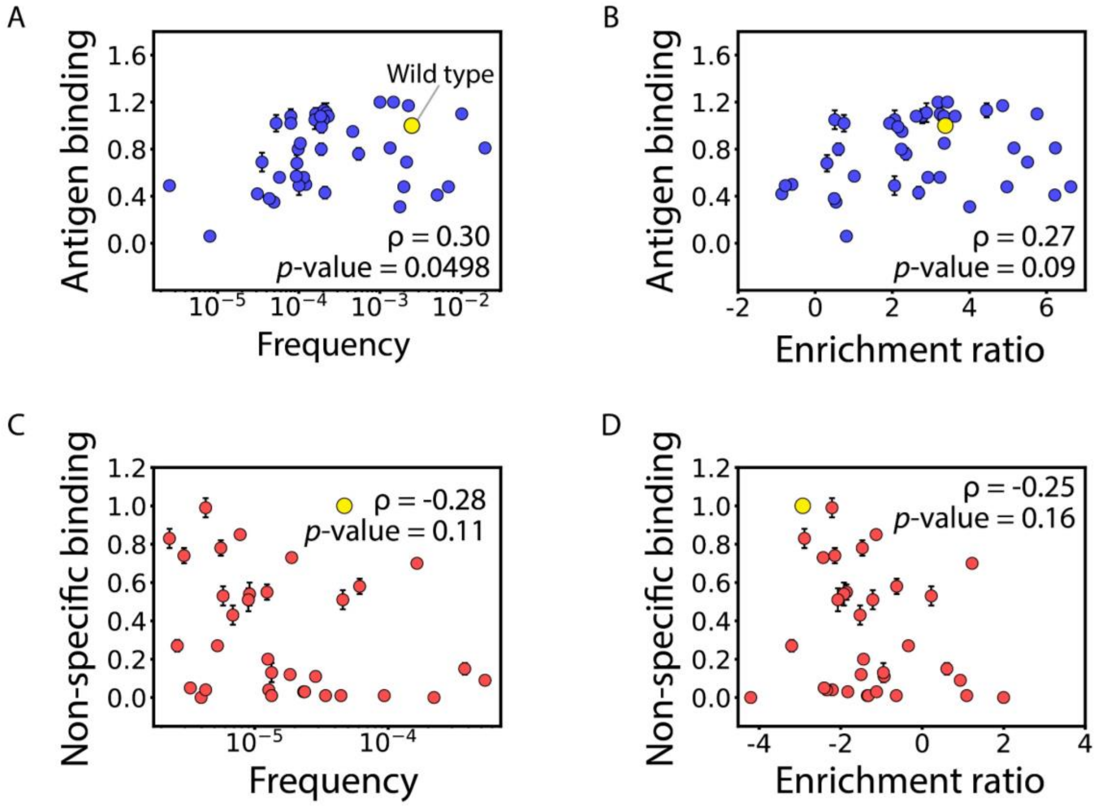

Figure S6. Conventional analysis of the deep sequencing data is generally poorly predictive of antibody IgG mutants with either improved affinity or specificity. (A-D) The deep sequencing datasets for the libraries enriched for high affinity and low non-specific binding (ovalbumin) were used to evaluate the (A, C) frequencies and (B, D) enrichment ratios of each antibody mutant, and the values were correlated with the experimental measurements as soluble IgGs. The experimental measurements were performed three times and the average values are reported. In (A-D), the experimental measurements are averages of three independent experiments, and the error bars are standard deviations. Independent two-sided *t*-tests were performed to determine significance.

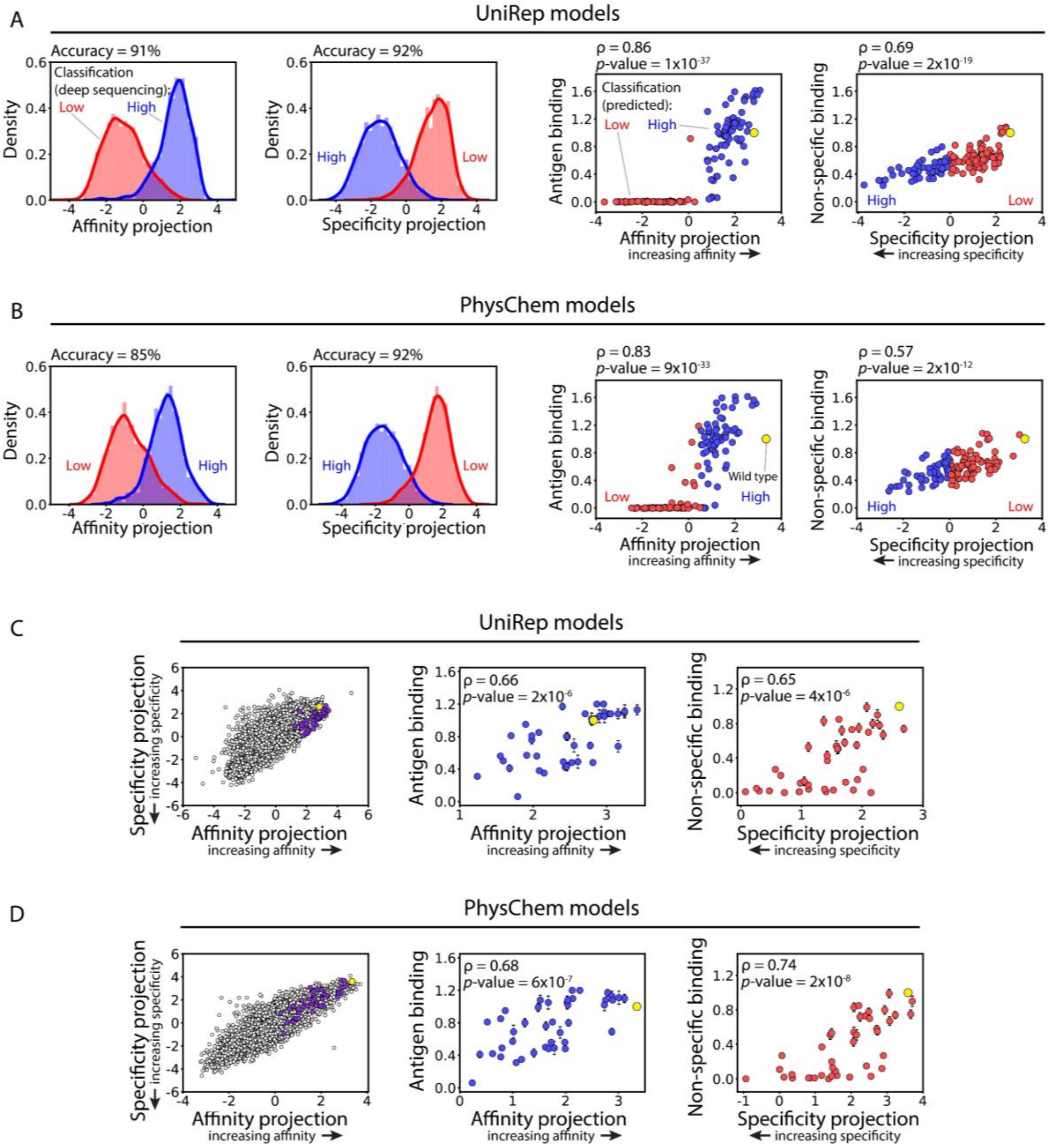

Figure S7. LDA models trained using deep learning and physicochemical features accurately classify antibody mutants with high and low levels of affinity and specificity and predict intraclass variability. (A-B) LDA models were trained using (A) deep learning (UniRep) and (B) physicochemical (PhysChem) features and displayed high test accuracy for classifying antibody affinity and specificity for 4000 antibodies identified in the enriched libraries via deep sequencing. The continuous predictions of the LDA models are strongly correlated with relative affinity and non-specific binding for 125 single-chain antibodies (Fabs) selected from the sorted libraries. The experimental measurements were performed as described in Figure 3. (C-D) Visualization of the Pareto frontier for the 4000 library variants using the LDA projections based on (C) UniRep and (D) PhysChem features shown in (A) and (B). Antibodies near the Pareto frontier were generated as soluble IgGs, and their antigen and non-specific binding levels were measured and plotted against model projections, as shown for LDA models trained with OneHot features in Figure 4. In (C-D), the experimental measurements are averages of three independent experiments, and the error bars are standard deviations. Independent two-sided *t*-tests were performed to determine significance.

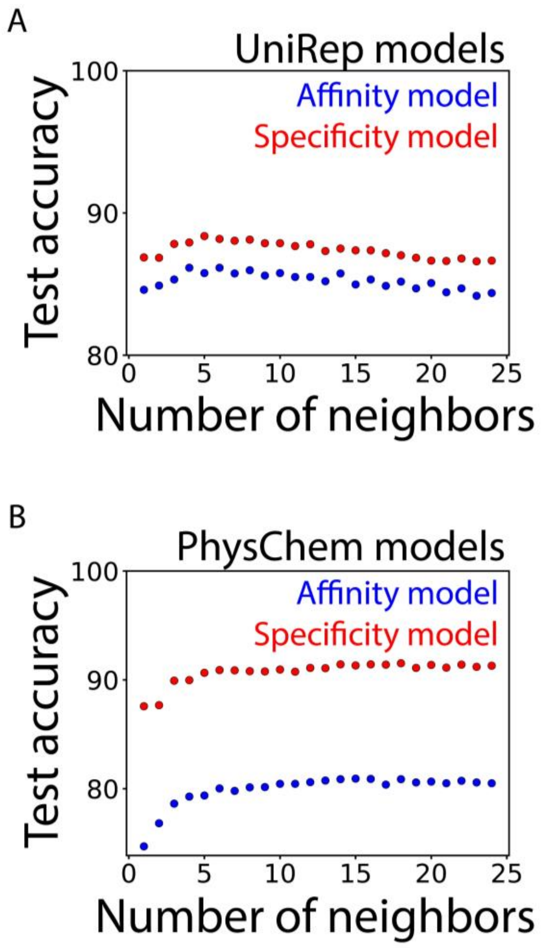

Figure S8. K-Nearest Neighbors (KNN) classification of antibody affinity and specificity using UniRep and PhysChem features. KNN models were trained for classifying antibody affinity and specificity (4000 antibodies) using (A) UniRep features and (B) PhysChem features. Classification accuracies for a range of k values were evaluated using 5-fold cross validation, with average test accuracies shown. Overall model accuracies of 86%, 79% (affinity) and 88%, 91% (specificity) were achieved using full datasets and number of neighbors = 5 for the UniRep and PhysChem models, respectively.

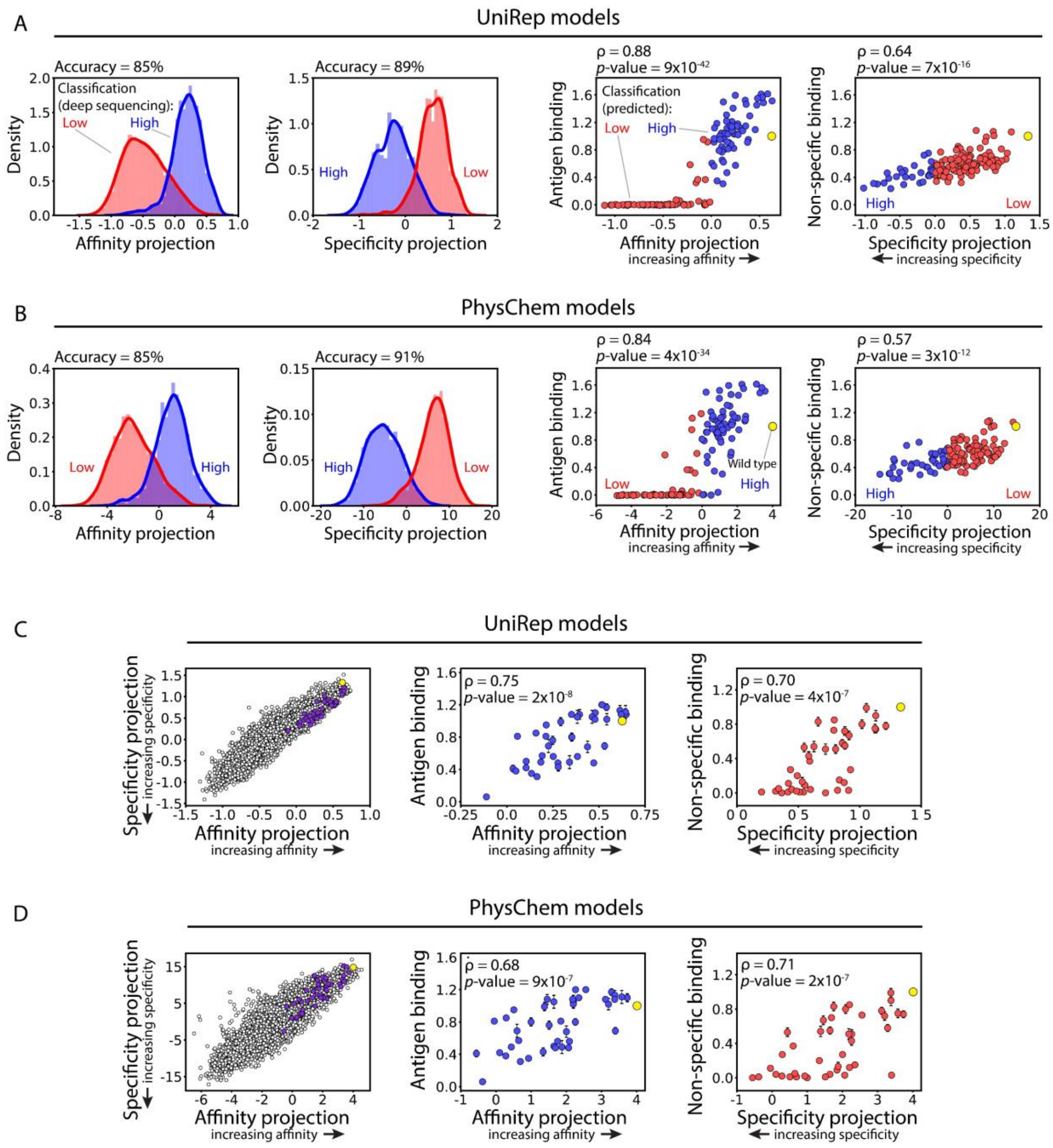

Figure S9. Neural network models trained using deep learning and physicochemical features accurately classify antibody mutants with high and low levels of affinity and specificity and predict intraclass variability. Neural network models (described in Figure S5) trained on deep learning (UniRep) and physicochemical (PhysChem) features achieve similar performance compared to LDA models. (A-B) Classification accuracy (deep sequencing) and correlation with experimental measurements for single-chain Fabs were evaluated for (A) deep learning and (B) physicochemical features, demonstrating similar performance compared to LDA models trained with the same features (Figure S7). (C-D) Antibodies near the Pareto frontier were generated as soluble IgGs, and their antigen and non-specific binding levels were measured and plotted against the model projections. In (C-D), the experimental measurements are averages of three independent experiments, and the error bars are standard deviations. Independent two-sided *t*-tests were performed to determine significance.

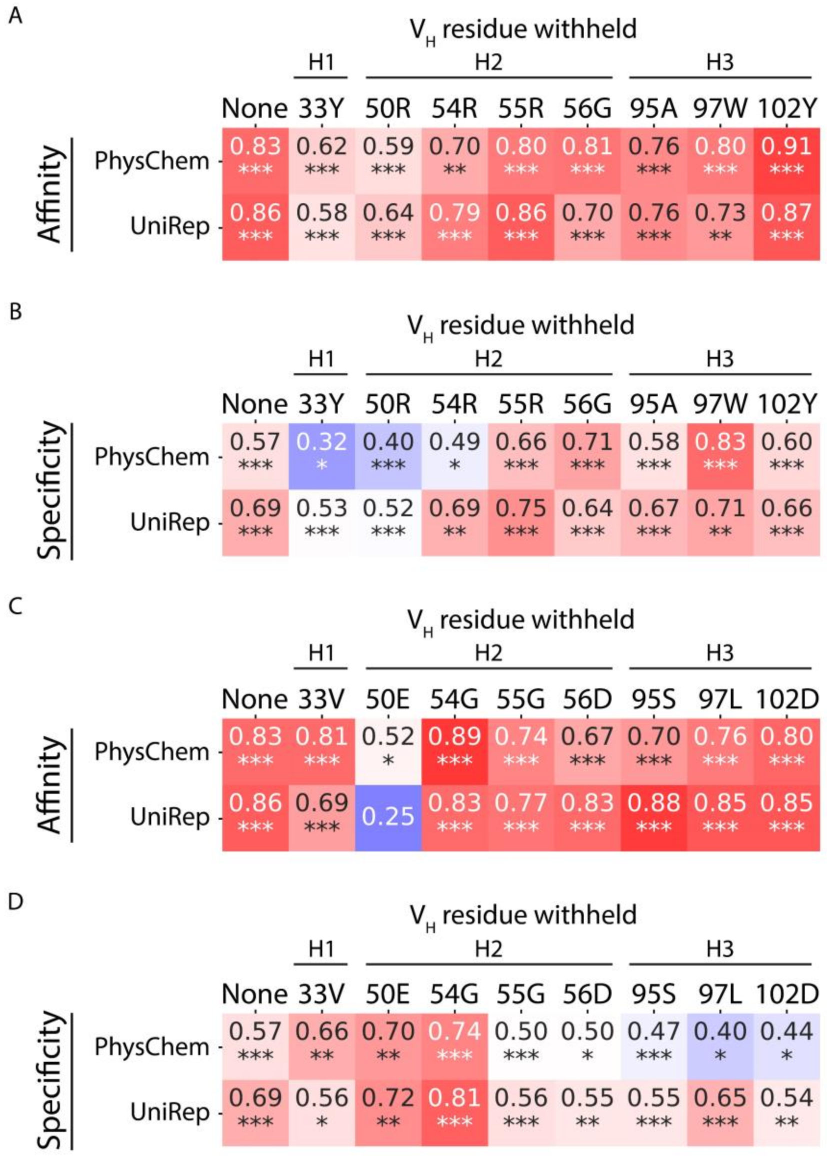

Figure S10. Evaluation of UniRep and PhysChem LDA models using leave-one-out analysis. (A-B) The models were trained on sets of antibodies (4,000 sequences) that lack information about a given wild-type residue at one of the eight mutated CDR sites (i.e., no antibodies with the wild-type residue at a given CDR site were included in the training process). Next, the trained models were tested on antibodies that have the corresponding wild-type CDR residue regardless of the identity of the CDR residues at the other seven mutated CDR sites. Finally, the Spearman's ρ values and *p*-values were evaluated for correlations between the experimental measurements of antibody variants on yeast (as single chain Fabs) and the model projections for (A) affinity and (B) specificity. (C-D) The analysis in (A-B) was repeated for the most common non-wild-type residue at each CDR position for (C) affinity and (D) specificity in order to test the generality of the leave-one-out predictions. Independent two-sided *t*-tests were performed to determine significance and the reported *p*-values were < 0.05 (*), <0.01 (**) and <0.001 (***).

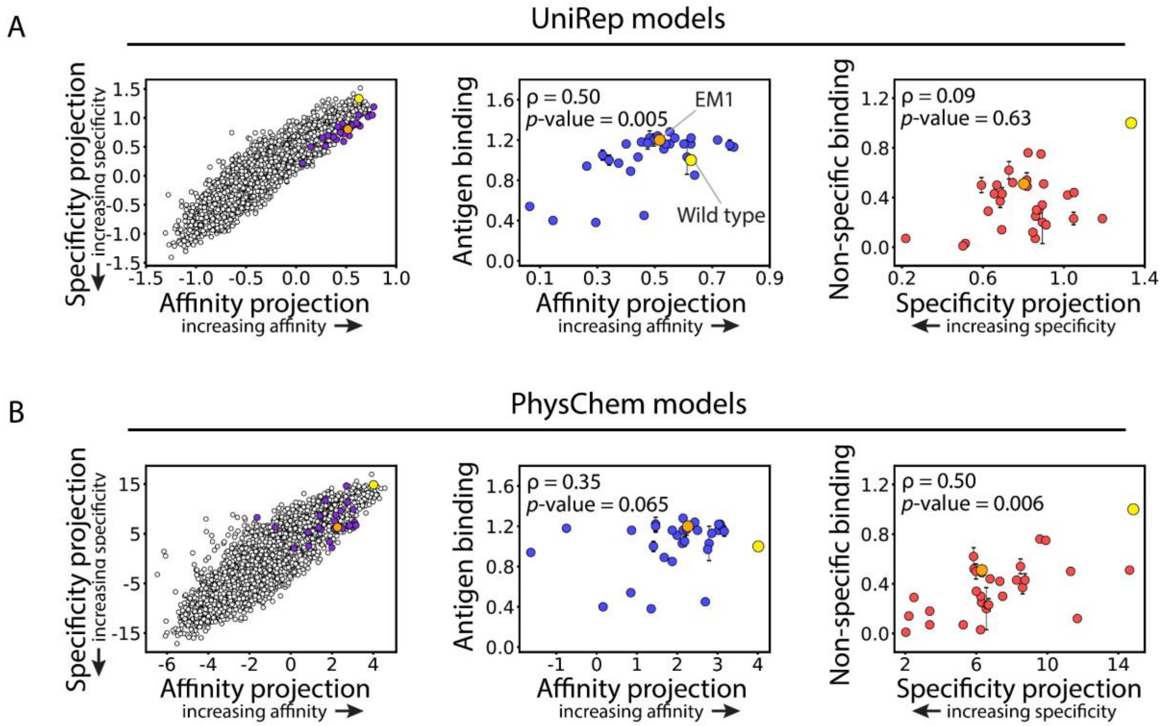

Figure S11. Evaluation of the predictions of neural network models for generalizing to novel mutational space. (A-B) Neural networks were trained with (A) deep learning (UniRep) and (B) physicochemical (PhysChem) features and used to predict novel mutations absent from the initial libraries, as shown in Figure 5 for the LDA models. Antibodies near the Pareto frontier (based on analysis from Figure 5) were generated as soluble IgGs, and their antigen and non-specific binding levels were measured and plotted against the model projections. In (A-B), the experimental measurements are averages of three independent experiments, and the error bars are standard deviations. Independent two-sided *t*-tests were performed to determine significance.

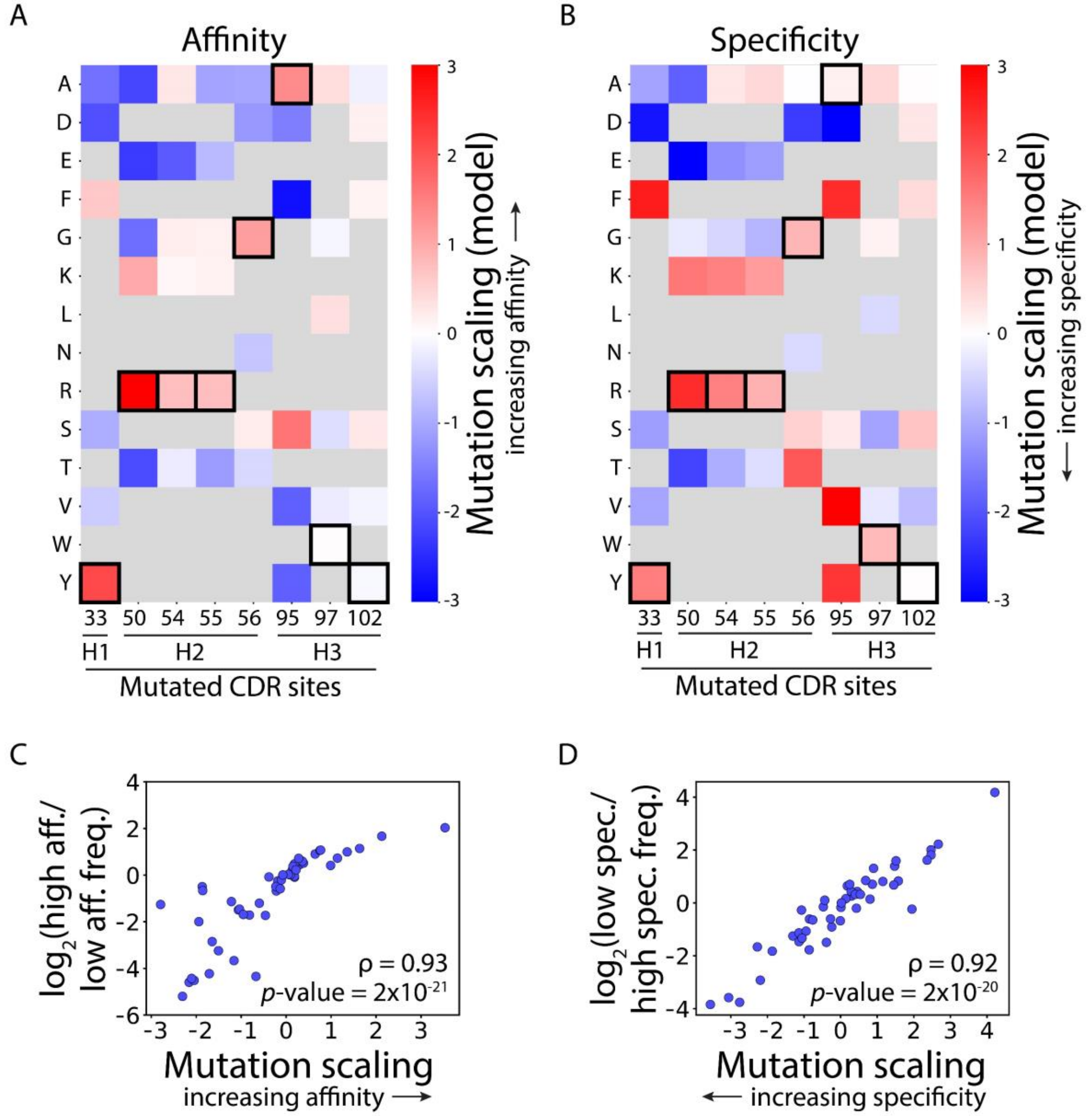

Figure S12. OneHot model scaling parameters are strongly correlated with the ratio of mutational frequencies in the high affinity and high non-specific binding library samples relative to the low affinity and low non-specific binding library samples. (A, B) The OneHot scaling parameters for the (A) affinity and (B) specificity models. (C, D) Correlation between the mutational scaling parameters and the ratio of mutational frequencies in the library samples selected for high affinity and high non-specific binding relative to those selected for low affinity and low non-specific binding. In (A) and (B), the black boxes signify the wild-type residues. Independent two-sided *t*-tests were performed to determine significance.

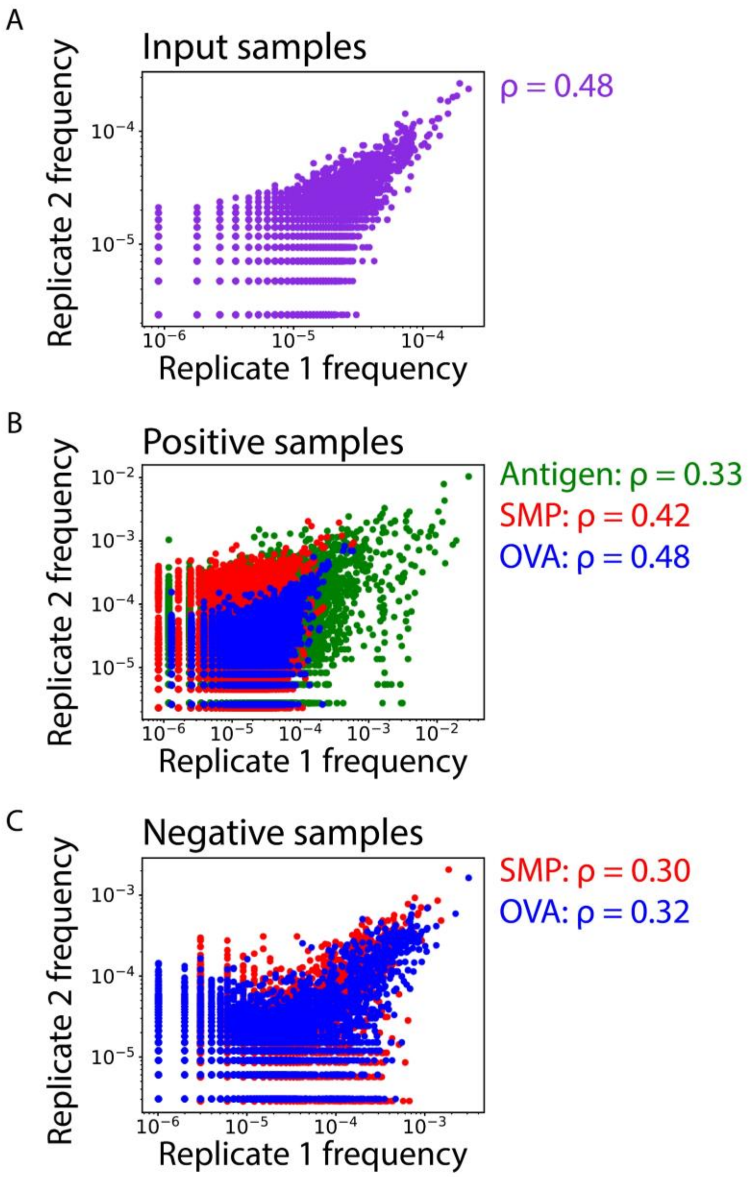

Figure S13. Deep sequencing antibody frequencies are correlated between replicates. The frequency of individual antibody sequences in each deep sequencing replicate were correlated for the (A) input samples, (B) antigen, soluble membrane proteins (SMP) and ovalbumin (OVA) positive samples, and (C) SMP and OVA negative samples. Spearman's ρ values are reported.

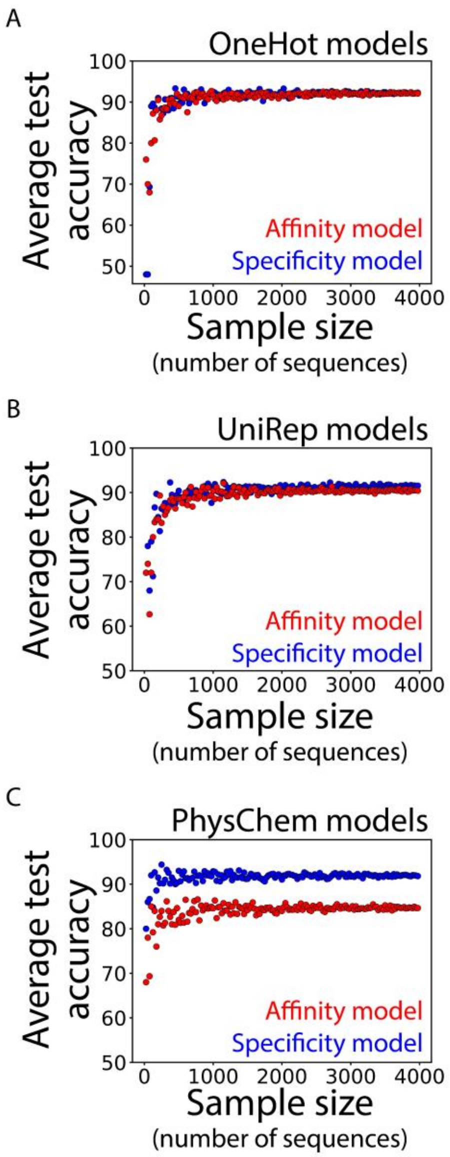

Figure S14. Impact of sample size on LDA model training accuracy. Fivefold cross-validation accuracies were evaluated for LDA models trained on antibody deep sequencing sets of different sizes for (A) OneHot, (B) deep learning (UniRep), and (C) physicochemical (PhysChem) features.

References

1 Kyte, J. & Doolittle, R. F. A simple method for displaying the hydropathic character of a protein. *J. Mol. Biol.* **157**, 105-132, doi:10.1016/0022-2836(82)90515-0 (1982).

2 Zhang, Y. *et al.* Physicochemical Rules for Identifying Monoclonal Antibodies with Drug-like Specificity. *Mol Pharm* **17**, 2555-2569, doi:10.1021/acs.molpharmaceut.0c00257 (2020).
