# Supplementary material for: Co-optimization of therapeutic antibody affinity and specificity using machine learning models that generalize to novel mutational space
Source: Nat Commun. 2022 Jul 1;13:3788. doi: 10.1038/s41467-022-31457-3 (PMC9249733; doi:10.1038/s41467-022-31457-3)
Supplement: Supplementary file 2 — Reporting Summary [file 41467_2022_31457_MOESM2_ESM.pdf]

nature portfolio

Corresponding author(s): Peter M. Tessier

Last updated by author(s): May 31, 2022

Reporting Summary

Nature Portfolio wishes to improve the reproducibility of the work that we publish. This form provides structure for consistency and transparency in reporting. For further information on Nature Portfolio policies, see our Editorial Policies and the Editorial Policy Checklist.

Statistics

For all statistical analyses, confirm that the following items are present in the figure legend, table legend, main text, or Methods section.

n/a	Confirmed	
☐	☒	The exact sample size (*n*) for each experimental group/condition, given as a discrete number and unit of measurement
☐	☒	A statement on whether measurements were taken from distinct samples or whether the same sample was measured repeatedly
☐	☒	The statistical test(s) used AND whether they are one- or two-sided<br>*Only common tests should be described solely by name; describe more complex techniques in the Methods section.*
☒	☐	A description of all covariates tested
☒	☐	A description of any assumptions or corrections, such as tests of normality and adjustment for multiple comparisons
☐	☒	A full description of the statistical parameters including central tendency (e.g. means) or other basic estimates (e.g. regression coefficient) AND variation (e.g. standard deviation) or associated estimates of uncertainty (e.g. confidence intervals)
☐	☒	For null hypothesis testing, the test statistic (e.g. *F*, *t*, *r*) with confidence intervals, effect sizes, degrees of freedom and *P* value noted<br>*Give P values as exact values whenever suitable.*
☒	☐	For Bayesian analysis, information on the choice of priors and Markov chain Monte Carlo settings
☒	☐	For hierarchical and complex designs, identification of the appropriate level for tests and full reporting of outcomes
☒	☐	Estimates of effect sizes (e.g. Cohen's *d*, Pearson's *r*), indicating how they were calculated

Our web collection on statistics for biologists contains articles on many of the points above.

Software and code

Policy information about availability of computer code

Data collection	No software was used for data collection in this study.
Data analysis	All software used to analyze data is described in the Methods and will be provided online<br>Sklearn was used to build models in python (version 3.8.4)<br>Scipy was used for statistical analysis (version 1.7.3)<br>Matplotlib (version 3.4.3) and seaborn (version 0.11.2) were used to generate figures<br>Paratope analysis was performed with Parapred (https://github.com/eliberis/parapred)<br>Antibody homology modeling was performed using Molecular Operating Environment (version 2021.05)<br>Homology model visualization was performed using Pymol (version 2.4)

For manuscripts utilizing custom algorithms or software that are central to the research but not yet described in published literature, software must be made available to editors and reviewers. We strongly encourage code deposition in a community repository (e.g. GitHub). See the Nature Portfolio guidelines for submitting code & software for further information.

Data

Policy information about availability of data

All manuscripts must include a data availability statement. This statement should provide the following information, where applicable:

- Accession codes, unique identifiers, or web links for publicly available datasets
- A description of any restrictions on data availability
- For clinical datasets or third party data, please ensure that the statement adheres to our policy

All data reported in the main text are available with the paper, at BioProject (Accession code: PRJNA850089), at Sequencing Read Archive (Accession codes:

SRX15766900 - SRX15766911), and at github.com/Tessier-Lab-UMich/Emi_Pareto_Opt_ML. Structure files for homology models were acquired from the protein data bank (PDB).

Field-specific reporting

Please select the one below that is the best fit for your research. If you are not sure, read the appropriate sections before making your selection.

☒ Life sciences ☐ Behavioural & social sciences ☐ Ecological, evolutionary & environmental sciences

For a reference copy of the document with all sections, see nature.com/documents/nr-reporting-summary-flat.pdf

Life sciences study design

All studies must disclose on these points even when the disclosure is negative.

Sample size	No methods were used to determine the sample size. Dataset sizes were constrained by the amount of resources required to generate samples. Within those constraints, datasets were selected based on the authors discretion to adequately demonstrate experimental outcomes.
Data exclusions	Measurements of sequences with novel mutations with low conservation scores (Blosum62) are excluded from the novel mutation analysis. This is described in detail in the Results and Methods.
Replication	All independent replicate measurements were successful. Most data was collected in independent biological triplicate to ensure reproducibility. Single-point yeast binding strengths (Figure 3C-D) were measured in duplicate due to the resource intensive nature of the experimentation.
Randomization	ScFab sequences chosen for binding measurements on yeast were selected randomly from propagated library selections. No other randomization was necessary as we performed quantitative in vitro experiments.
Blinding	No blinding was included in this study. Computational data analysis was automated and unbiased.

Reporting for specific materials, systems and methods

We require information from authors about some types of materials, experimental systems and methods used in many studies. Here, indicate whether each material, system or method listed is relevant to your study. If you are not sure if a list item applies to your research, read the appropriate section before selecting a response.

Materials & experimental systems

n/a	Involved in the study	
☐	☒	Antibodies
☐	☒	Eukaryotic cell lines
☒	☐	Palaeontology and archaeology
☒	☐	Animals and other organisms
☒	☐	Human research participants
☒	☐	Clinical data
☒	☐	Dual use research of concern

Methods

n/a	Involved in the study	
☒	☐	ChIP-seq
☐	☒	Flow cytometry
☒	☐	MRI-based neuroimaging

Antibodies

Antibodies used	Cell signaling technologies: mouse anti-Myc mAb (2276S)<br>Jackson ImmunoResearch: goat anti-human AF647 (109-605-098), goat anti-human Fc (109-005-008)<br>Life Technologies: goat anti-mouse AF488 (A11001)<br>Invitrogen: goat anti-human Fc F(ab')2 AF-488 (H10120)
Validation	All antibodies used are commercially available and widely used. We refer to the manufacturers statements on validation:<br><br>Cell signaling technologies - Mouse anti-Myc mAb: Validated according to CST's Hallmarks of antibody validation (https://www.cellsignal.com/about-us/cst-antibody-validation-principles).<br><br>Jackson ImmunoResearch - goat anti-human AF647 and goat anti-human Fc: Validated using immunoelectrophoresis or ELISA (https://www.jacksonimmuno.com/catalog/products/109-001-008 and https://www.jacksonimmuno.com/catalog/products/109-605-003)<br><br>Life Technologies/invitrogen - goat anti-mouse AF488 and goat anti-human Fc F(ab')2 AF-488: Validation information at https://www.thermofisher.com/us/en/home/life-science/antibodies/invitrogen-antibody-validation.html with product information at

https://www.thermofisher.com/antibody/product/Goat-anti-Mouse-IgG-H-L-Secondary-Antibody-Recombinant-Polyclonal/A28175

Eukaryotic cell lines

Policy information about cell lines

Cell line source(s)	NCI H596 cells were obtained from ATCC (HTB-178)<br>HEK293EBNA1-6E cells were obtained from National Research Council Canada (L-11565)
Authentication	Cell lines were authenticated by the providers. NCI H596 cells were authenticated using short tandem repeat analysis. HEK293EBNA1-6E are proprietary.
Mycoplasma contamination	Cells were not tested for mycoplasma.
Commonly misidentified lines<br>(See ICLAC register)	No commonly misidentified cell lines were used.

Flow Cytometry

Plots

Confirm that:

- [x] The axis labels state the marker and fluorochrome used (e.g. CD4-FITC).
- [x] The axis scales are clearly visible. Include numbers along axes only for bottom left plot of group (a 'group' is an analysis of identical markers).
- [x] All plots are contour plots with outliers or pseudocolor plots.
- [x] A numerical value for number of cells or percentage (with statistics) is provided.

Methodology

Sample preparation	Reagents were incubated with the yeast cells for 3 h at room temperature. Soluble membrane proteins were incubated with yeast cells for 20 min on ice. A mouse anti-Myc mAb was co-incubated with the antigen and polyspecificity reagents to evaluate antibody display. The bound reagents were detected using different secondary reagents. The biotinylated polyspecificity reagents were detected using streptavidin AF647. The antigen was detected with goat anti-human AF647. The anti-Myc tag antibody was detected using goat anti-mouse AF488.
Instrument	Cells were sorted on a Beckman-Coulter MoFlo Astrios sorter and analyzed on a BioRad Zeti5 analyzer.
Software	No additional software was used to analyze the flow data.
Cell population abundance	Cell sorting was performed on a minimum of 10^7 cells. Analysis was performed on a minimum of 50,000 cells.
Gating strategy	Sorting gates were designed to collect the top and bottom 10% of binders in each experiment. Boundaries were drawn on the diagonal through the displaying population to account for higher density of displaying antibodies on the surface.

- [x] Tick this box to confirm that a figure exemplifying the gating strategy is provided in the Supplementary Information.
